# Evaluation of anemia in non-enhanced and contrast-enhanced dual-energy CT using electron density imaging

**DOI:** 10.1371/journal.pone.0352504

**Published:** 2026-07-02

**Authors:** Cherry Kim, Suk Keu Yeom, Hangseok Choi, Euddeum Shim, Sung Ho Hwang, Hwan Seok Yong, Marly van Assen, Carlo N. De Cecco, Hwa Jung Sung, Se Ryeon Lee

**Affiliations:** 1 Department of Radiology and Research Institute of Radiological Science, Severance Hospital, Yonsei University College of Medicine, Seoul, Korea; 2 Department of Radiology, Ansan Hospital, Korea University College of Medicine, Ansan-si, Gyeonggi-do, South Korea; 3 Medical Science Research Center, Korea University College of Medicine, Goryeodae-ro, Seongbuk-gu, South Korea; 4 Department of Radiology, Anam Hospital, Korea University College of Medicine, Seoul, South Korea; 5 Department of Radiology, Guro Hospital, Korea University College of Medicine, Seoul, South Korea; 6 Department of Radiology and Imaging Sciences, Emory University School of Medicine, Atlanta, Georgia, United States of America; 7 Translational Laboratory for Cardiothoracic Imaging and Artificial Intelligence, Emory University School of Medicine, Atlanta, Georgia, United States of America; 8 Division of Cardiothoracic Imaging, Department of Radiology, Emory University, Atlanta, Georgia, United States of America; 9 Department of Internal Medicine, Ansan Hospital, Korea University College of Medicine, Ansan-si, Gyeonggi-do, South Korea; Universiti Malaysia Sabah, MALAYSIA

## Abstract

Electron density (ED) imaging derived from dual-energy CT (DECT) quantifies tissue composition and may reflect changes in red blood cell (RBC) mass associated with anemia. This study aimed to evaluate the utility of ED from DECT in assessing anemia severity using both non-enhanced CT (NECT) and contrast-enhanced CT (CECT), and to investigate its correlation with hemoglobin (Hb), hematocrit (Hct), and RBC count. In this retrospective study, 2,558 patients who underwent NECT and 2,545 who underwent CECT using a dual-layer spectral detector CT (Philips IQon) were included. Mean ED and mean CT attenuation (HU) were measured at five cardiac blood pool sites. Spearman’s rank correlation analysis was performed between CT measurements and hematologic parameters. Partial correlation analysis adjusting for age and sex and non-parametric bootstrap internal validation were also conducted. Receiver operating characteristic (ROC) curve analysis was performed to determine diagnostic cutoff values for anemia detection. Mean ED significantly decreased with increasing anemia severity in both NECT and CECT cohorts (all *p* < 0.001). With NECT, mean ED showed positive correlations with Hb, Hct, and RBC count (r_s_ = 0.726–0.770), with AUCs of 0.825–0.956 for anemia detection. With CECT, mean ED retained positive correlations with hematologic parameters (r_s_ = 0.447–0.583), with AUCs of 0.727–0.895, while mean HU showed no meaningful correlation. After adjustment for age and sex, partial correlation coefficients remained substantial (NECT: r_s_ = 0.694; CECT: r_s_ = 0.509 for Hb), and bootstrap internal validation confirmed negligible overfitting bias. ED derived from DECT demonstrated diagnostic utility for anemia detection on NECT, unlike conventional HU, retained clinically relevant associations with hematologic parameters on CECT. ED-based anemia detection on CECT is best conceptualized as opportunistic detection, particularly for severe anemia, supplementary to laboratory testing. These findings require external validation across diverse DECT platforms before broader clinical implementation.

## Introduction

Electron density (ED) imaging, derived from dual-energy CT, quantifies the probability of an electron being present in a specific tissue location and is highly sensitive to changes in tissue composition, particularly in molecular structure and cellularity. A recent study demonstrates that variations in ED values on non-enhanced CT (NECT) are closely linked to the composition and density of blood—acute pulmonary thromboemboli, which are rich in erythrocytes (and thus hemoglobin), consistently showed higher ED values than subacute and chronic thrombi where hemoglobin (Hb) content diminishes as water content increases [1].

Since anemia fundamentally reflects a reduction in Hb concentration and red cell mass [2,3], it follows that ED imaging may be able to non-invasively detect or quantify degrees of anemia by observing corresponding reductions in blood ED values, similar to how it tracks changes in clot composition. The ability to evaluate anemia through medical imaging, such as ED imaging from dual-energy CT, would offer significant clinical benefits by enabling the diagnosis of anemia in patients not initially suspected of having the condition. Several studies have explored the use of CT scans to evaluate anemia.

The relatively high attenuation of blood in CT imaging—attributed to RBCs carrying Hb—has been exploited in the use of NECT to evaluate anemia. In anemic patients, the concentration of red blood cells (RBCs) (and thus blood attenuation) is reduced, while the attenuation of cardiac muscle remains only slightly altered. Consequently, the interventricular septum becomes discernible [4–7]. Additionally, several studies have reported a linear correlation between the attenuation (Hounsfield Unit, HU) of the blood pool and serum Hb/hematocrit (Hct) levels with NECT [6,8–11]. In contrast-enhanced CT (CECT), the attenuation of the blood pool is predominantly determined by the concentration of contrast material due to its substantial effect. Consequently, changes in CT attenuation of the blood pool are less detectable with CECT. However, recent studies have introduced virtual non-contrast (VNC) reconstruction from dual-energy CT (DECT) or photon-counting CT to address this limitation, demonstrating that anemia can be detected and quantified on CECT [12–14].

Therefore, if ED proves useful in evaluating anemia even with CECT, it would be highly valuable. This study aimed to assess the utility of ED from DECT in the evaluation of anemia using both NECT and CECT and to investigate the correlation between ED and Hb, Hct, or RBC counts.

## Materials and methods

### Study design

**Fig 1:**
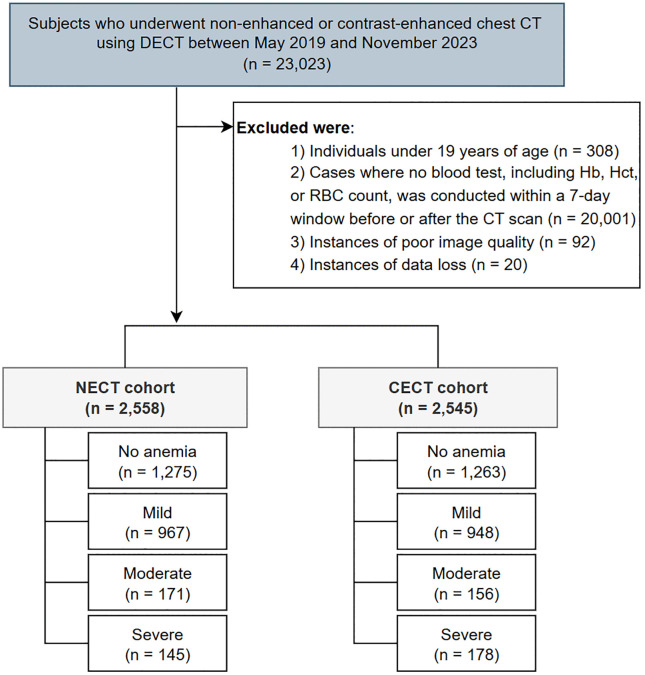
Flowchart depicting the recruitment and selection process. Anemia was defined as Hb levels < 13.0 g/dL in males and <12.0 g/dL in females. It was further categorized as mild (11.0–12.9 g/dL in males and 11.0–11.9 g/dL in females), moderate (8.0–10.9 g/dL), and severe (Hb < 8.0 g/dL) [3].

This retrospective study was approved by the institutional ethics review board (approval number: 2024AS0179), which granted a waiver of informed consent. No minors participated in the study. We identified 23,023 consecutive patients in our institution’s database who underwent either non-enhanced or contrast-enhanced chest CT using DECT between May 2019 and November 2023. The inclusion criteria were: 1) adults aged > 19 years because pediatric patients were excluded to avoid age-related differences in hematologic reference ranges and contrast enhancement characteristics; 2) individuals diagnosed with anemia or healthy individuals with normal Hb levels; and 3) those with Hb, Hct, and RBC count tests performed within 7 days before or after the CT scan. Exclusion criteria included poor image quality or data loss preventing the reconstruction of ED maps (Fig 1). The data were accessed for research purposes from July 24^th^, 2024, for a period of six months, and the authors did not have access to any information that could identify individual participants during or after data collection.

### CT protocol

All patients were scanned using a Philips IQon 128-slice dual-layer detector spectral CT scanner (Philips Healthcare, Cleveland, OH, USA). The CT scan parameters are detailed in S1 Table. For CECT, an intravenous contrast medium was administered at a rate of 3.5 mL/s via a 20-gauge cannula into the antecubital vein using a power injector. CT scans were obtained 30 seconds after intravenous contrast administration in all patients. The protocols employed for CECT were consistent across all patients. Spectral base images (SBI) were reconstructed for all patients and included conventional CT with basis images. Using SBI, ED maps were generated.

### Measurement of HU and ED

**Fig 2:**
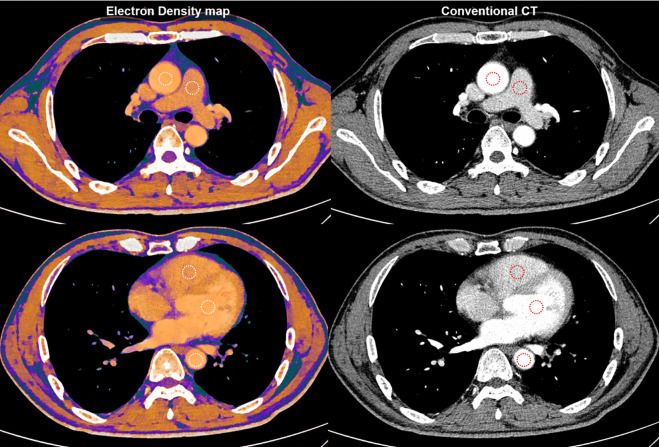
ROI placement on conventional CT and ED map. ROIs are drawn on the ascending aorta and pulmonary trunk at the level of the pulmonary trunk, and on the right ventricle, left ventricle, and descending aorta at the level of the left ventricle.

All images were automatically transmitted to a post-processing workstation (ISP, version 10, Philips Healthcare, Cleveland, OH, USA) for evaluation. Regions of interest (ROIs) with an approximate area of 2 cm^2^ were drawn by two thoracic radiologists with 4 and 11 years of experience (BLINDED-FOR-REVIEW). They analyzed both conventional CT scans and ED maps of NECT and CECT scans to measure HU and ED values in consensus. ROIs were placed in the ascending aorta and pulmonary trunk at the level of the pulmonary trunk, and in the right ventricle, left ventricle, and descending aorta at the level of the left ventricle (Fig 2). All measured values were averaged (mean HU and mean ED). The ED value was expressed as a percentage relative to the ED of water (%EDW).

### Statistical analysis

Statistical analysis was conducted using R software (version 4.3.2; R Foundation for Statistical Computing, Vienna, Austria). A P-value of less than 0.05 was considered statistically significant. The Shapiro-Wilk test evaluated the normality of the distribution. For continuous parameters, one-way analysis of variance (ANOVA) or the Kruskal-Wallis test was employed, with multiple comparison methods such as the Wilcoxon rank-sum test or the two-sample t-test used to compare differences among variables, with Bonferroni-adjusted P-values. Correlation analysis was performed to evaluate the associations between CT-derived measurements and Hb, Hct, and RBC count levels in order to assess validity. When at least one of the two variables did not satisfy the assumption of normality, Spearman’s rank correlation coefficient (*r*_*s*_) was used. When both variables met the assumption of normality, Pearson’s product–moment correlation coefficient was applied. Correlation coefficients greater than 0.8 were deemed strong, 0.8–0.5 moderate, 0.5–0.3 weak, and less than 0.3 negligible. To determine the optimal diagnostic parameter, scatter plots and simple linear regression analysis were conducted within the study groups. Receiver operating characteristic (ROC) curve analysis was performed to establish threshold values for diagnosing anemia or severe anemia. To adjust for potential demographic confounding, partial correlation analysis controlling for age and sex was performed using Spearman’s rank partial correlation coefficient. To assess the proportion of variance in hematologic parameters explained by mean ED and mean HU after demographic adjustment, the coefficient of determination (R²) was calculated as the square of the partial Spearman’s rank correlation coefficient. To evaluate the internal validity and stability of the partial correlation estimates and to assess for overfitting, nonparametric bootstrap resampling was performed with 1,000 iterations using resampling with replacement. Bootstrap bias and standard error were estimated for each partial correlation coefficient, and 95% bootstrap confidence intervals were derived.

## Results

### Baseline characteristics

The NECT cohort (n = 2,558) comprised 1,275 subjects without anemia (510 males/765 females), 967 with mild anemia (559 males/408 females), 171 with moderate anemia (88 males/83 females), and 145 with severe anemia (46 males/99 females), with mean ages of 58.05 ± 13.36, 63.70 ± 13.81, 64.20 ± 13.91, and 62.81 ± 14.37 years, respectively. The CECT cohort (n = 2,545) included 1,263 subjects without anemia (503 males/760 females), 948 with mild anemia (548 males/400 females), 156 with moderate anemia (83 males/73 females), and 178 with severe anemia (58 males/120 females), with mean ages of 58.00 ± 13.33, 63.74 ± 13.80, 64.67 ± 13.58, and 62.01 ± 15.43 years, respectively (Table 1).

**Table 1:** Baseline characteristics and laboratory findings of non-enhanced CT and contrast-enhanced CT cohorts.

NECT cohort (n = 2,558)	No anemia (n = 1,275)	Mild (n = 967)	Moderate (n = 171)	Severe (n = 145)
Age (years)	58.05 ± 13.36	63.70 ± 13.81	64.20 ± 13.91	62.81 ± 14.37
Sex (%)				
Male (n = 1,203)	510 (42.4)	559 (46.5)	88 (7.3)	46 (3.8)
Female (n = 1,355)	765 (56.5)	408 (30.1)	83 (6.1)	99 (7.3)
Hb (g/dL)	14.08 ± 1.18	11.81 ± 1.17	8.78 ± 0.45	7.20 ± 0.76
Hct (%)	42.65 ± 3.36	36.34 ± 3.51	27.51 ± 2.02	22.52 ± 2.63
RBC count (per pL)	4.63 ± 0.43	3.93 ± 0.49	3.02 ± 0.49	2.49 ± 0.52
**CECT cohort (n = 2,545)**	**No anemia (n = 1,263)**	**Mild (n = 948)**	**Moderate (n = 156)**	**Severe (n = 178)**
Age (years)	58.00 ± 13.33	63.74 ± 13.80	64.67 ± 13.58	62.01 ± 15.43
Sex (%)				
Male (n = 1,192)	503 (42.2)	548 (46.0)	83 (7.0)	58 (4.9)
Female (n = 1,353)	760 (56.2)	400 (29.6)	73 (5.4)	120 (8.9)
Hb (g/dL)	14.07 ± 1.18	11.83 ± 1.17	8.79 ± 0.44	7.01 ± 0.89
Hct (%)	42.64 ± 3.34	36.39 ± 3.49	27.63 ± 1.99	22.01 ± 3.11
RBC count (per pL)	4.63 ± 0.43	3.94 ± 0.48	3.06 ± 0.49	2.51 ± 0.58

NECT, non-enhanced CT; ED, electron density image; HU, CT attenuation; CECT, contrast-enhanced CT

### Quantitative CT analysis

**Fig 3:**
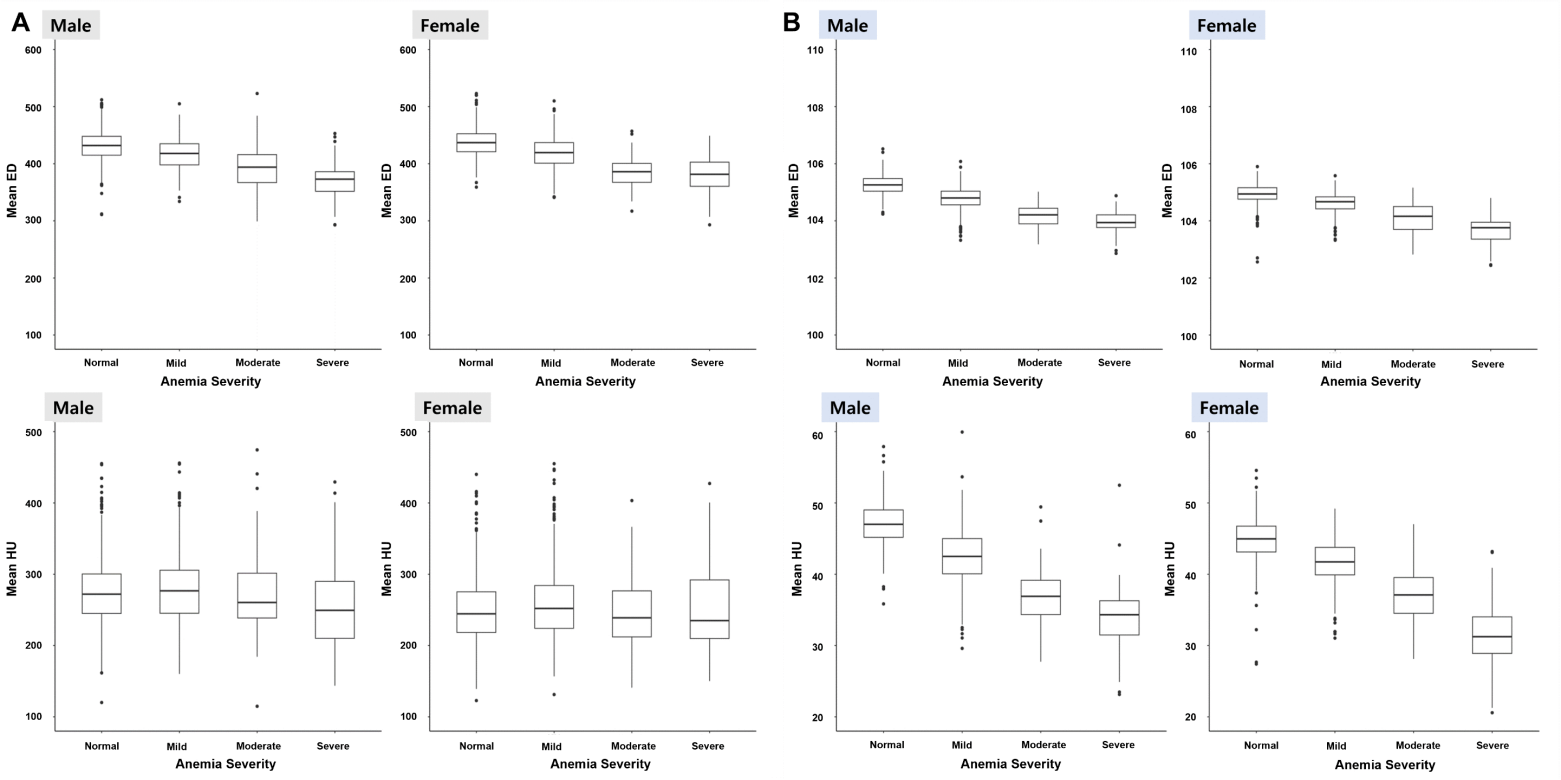
Boxplots of mean ED and HU across anemia severity groups. (A) contrast-enhanced CT and (B) non-enhanced CT.

In association with NECT, both the mean ED and mean HU demonstrated a significant decrease with increasing anemia severity in both male and female subjects (Table 2, S2, S3 Tables, and Fig 3): The mean ED values for no anemia, mild, moderate, and severe anemia were 105.25 ± 0.34, 104.78 ± 0.39, 104.15 ± 0.41, and 103.94 ± 0.45 in males, respectively, and 104.94 ± 0.34, 104.60 ± 0.36, 104.11 ± 0.50, and 103.65 ± 0.49 in females, respectively. These differences were all statistically significant (p < 0.001). The mean HU values for no anemia, mild, moderate, and severe anemia were 47.05 ± 2.90, 42.31 ± 3.67, 36.89 ± 3.74, and 33.74 ± 5.73 in males, respectively, and 44.94 ± 2.81, 41.75 ± 2.96, 37.15 ± 3.94, and 30.58 ± 5.61 in females, respectively. Both mean ED and mean HU varied significantly among the different anemia severity groups.

**Table 2:** Comparisons of mean HU and ED according to anemia severity in NECT and CECT cohorts.

NECT cohort (n = 2,558)		No anemia (n = 1,275)	Mild anemia (n = 967)	Moderate anemia (n = 171)	Severe anemia (n = 145)	p -value
**Mean ED**	**All patients**	105.06 ± 0.37	104.70 ± 0.39*	104.13 ± 0.46*†	103.74 ± 0.50*†§	<0.001
	**Male**	105.25 ± 0.34	104.78 ± 0.39*	104.15 ± 0.41*†	103.94 ± 0.45*†§	<0.001
	**Female**	104.94 ± 0.34	104.60 ± 0.36*	104.11 ± 0.50*†	103.65 ± 0.49*†§	<0.001
**Mean HU**	**All patients**	45.78 ± 3.03	42.07 ± 3.40*	37.02 ± 3.83*†	31.58 ± 5.81*†§	<0.001
	**Male**	47.05 ± 2.90	42.31 ± 3.67*	36.89 ± 3.74*†	33.74 ± 5.73*†§	<0.001
	**Female**	44.94 ± 2.81	41.75 ± 2.96*	37.15 ± 3.94*†	30.58 ± 5.61*†§	<0.001
**CECT cohort (n = 2,545)**		**No anemia (n = 1,263)**	**Mild anemia (n = 948)**	**Moderate anemia (n = 156)**	**Severe anemia (n = 178)**	**p -value**
**Mean ED**	**All patients**	106.67 ± 0.52	106.35 ± 0.55*	105.75 ± 0.68*†	105.46 ± 0.63*†§	<0.001
	**Male**	106.75 ± 0.52	106.38 ± 0.55*	105.69 ± 0.52*†	105.59 ± 0.64*†	<0.001
	**Female**	106.61 ± 0.52	106.32 ± 0.57*	105.83 ± 0.82*†	105.40 ± 0.62*†§	<0.001
**Mean HU**	**All patients**	265.42 ± 48.95	266.77 ± 51.05	262.31 ± 60.48	253.61 ± 58.90*†	<0.001
	**Male**	250.69 ± 47.86	258.33 ± 49.66	248.64 ± 49.92	253.15 ± 59.89	0.020
	**Female**	275.17 ± 47.22	278.32 ± 50.73	277.86 ± 67.64	253.84 ± 58.67*	<0.001

NECT, non-enhanced CT; ED, electron density image; HU, CT attenuation; CECT, contrast-enhanced CT

*p < 0.05 (vs. No anemia)

†p < 0.05 (vs. Mild anemia)

§p < 0.05 (vs. Moderate anemia)

With CECT, the mean ED for both males and females significantly decreased with increasing anemia severity (mean ED values for no anemia, mild, moderate, and severe anemia in males were 106.75 ± 0.52, 106.38 ± 0.55, 105.69 ± 0.52, and 105.59 ± 0.64, respectively; for females, 106.61 ± 0.52, 106.32 ± 0.57, 105.83 ± 0.82, and 105.40 ± 0.62, respectively; all p < 0.001). Additionally, the mean ED values differed significantly across anemia severity groups. However, the mean HU values for all groups in both males and females did not exhibit any decreasing trends with increasing anemia severity.

### Correlation with laboratory parameters

**Fig 4:**
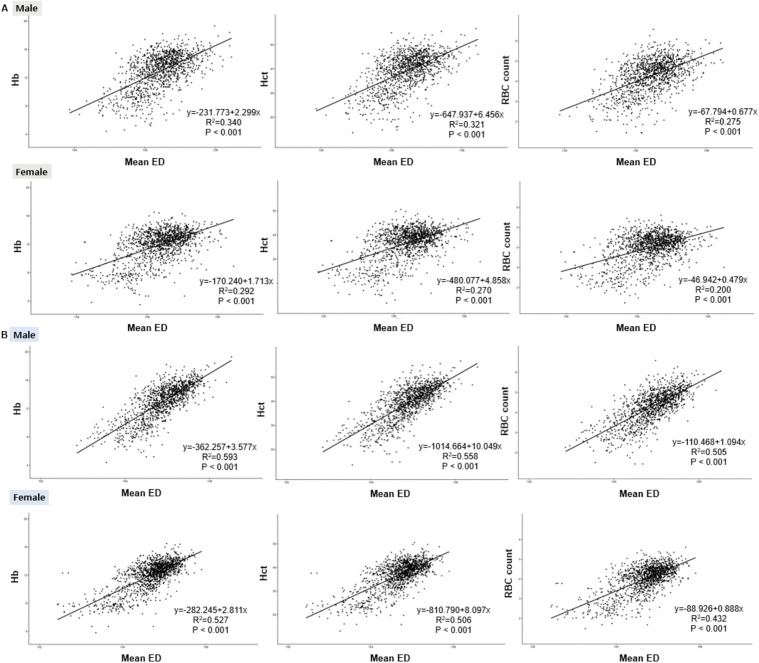
Scatter plots showing correlations between mean ED and laboratory parameters. (A) contrast-enhanced CT and (B) non-enhanced CT.

Correlations between CT measurements and Hb, Hct, and RBC count levels are shown in Table 3, Fig 4, and S1 Fig. With NECT, the mean ED exhibited strong positive correlations with Hb, Hct, and RBC count in both males and females (*r*_*s*_ for males: 0.770, 0.747, and 0.711; *r*_*s*_ for females: 0.726, 0.712, and 0.657; all p < 0.001). Similarly, in association with CECT, the mean ED also showed positive correlations with Hb, Hct, and RBC count in both sexes (*r*_*s*_ for males: 0.583, 0.566, and 0.525; *r*_*s*_ for females: 0.540, 0.520, and 0.447; all p < 0.001).

**Table 3:** Spearman’s rank correlations between CT measurements and laboratory findings.

NECT	Mean ED			Mean HU		
	All patients	Male	Female	All patients	Male	Female
***Hb***						
*r*_*s*_	0.756	0.770	0.726	0.789	0.801	0.784
Significant level	<0.001	<0.001	<0.001	<0.001	<0.001	<0.001
95% confidence interval	0.739 ~ 0.772	0.746 ~ 0.792	0.700 ~ 0.750	0.774 ~ 0.803	0.780 ~ 0.820	0.763 ~ 0.804
Formula to assess Hb	−326.55 + 3.24 x ED	−362.26 + 3.58 x ED	−282.25 + 2.81 x ED	−2.76 + 0.35 x ED	−4.05 + 0.39 x ED	−1.18 + 0.31 x ED
***Hct***						
*r*_*s*_	0.737	0.747	0.712	0.785	0.790	0.783
Significant level	<0.001	<0.001	<0.001	<0.001	<0.001	<0.001
95% confidence interval	0.719 ~ 0.754	0.721 ~ 0.771	0.684 ~ 0.737	0.770 ~ 0.800	0.768 ~ 0.811	0.761 ~ 0.803
Formula to assess Hct	−920.36 + 9.15 x ED	−1014.66 + 10.05 x ED	−810.79 + 8.10 x ED	−5.82 + 1.02 x ED	−9.28 + 1.12 x ED	−1.80 + 0.91 x ED
***RBC count***						
*r*_*s*_	0.694	0.711	0.657	0.733	0.747	0.716
Significant level	<0.001	<0.001	<0.001	<0.001	<0.001	<0.001
95% confidence interval	0.673 ~ 0.713	0.682 ~ 0.738	0.626 ~ 0.686	0.715 ~ 0.751	0.721 ~ 0.771	0.690 ~ 0.741
Formula to assess RBC count	−100.60 + 1.00 x ED	−110.47 + 1.09 x ED	−88.93 + 0.89 x ED	−0.62 + 0.11 x ED	−0.98 + 0.12 x ED	−0.20 + 0.10 x ED
**CECT**	**Mean ED**			**Mean HU**		
	**All patients**	**Male**	**Female**	**All patients**	**Male**	**Female**
***Hb***						
*r*_*s*_	0.556	0.583	0.540	−0.025	−0.018	0.054
Significant level	<0.001	<0.001	<0.001	0.21	0.536	0.047
95% confidence interval	0.529 ~ 0.582	0.544 ~ 0.619	0.501 ~ 0.577	N/A	N/A	N/A
Formula to assess Hb	−201.3 + 2.01 x ED	−231.77 + 2.3 x ED	−170.24 + 1.71 x ED	N/A	N/A	N/A
***Hct***						
*r*_*s*_	0.540	0.566	0.520	−0.019	−0.009	0.047
Significant level	<0.001	<0.001	<0.001	0.337	0.748	0.086
95% confidence interval	0.512 ~ 0.567	0.527 ~ 0.604	0.480 ~ 0.558	N/A	N/A	N/A
Formula to assess Hct	−563.92 + 5.66 x ED	−647.94 + 6.46 x ED	−480.08 + 4.86 x ED	N/A	N/A	N/A
***RBC count***						
*r*_*s*_	0.484	0.525	0.447	−0.059	−0.050	−0.001
Significant level	<0.001	<0.001	<0.001	0.003	0.083	0.984
95% confidence interval	0.454 ~ 0.513	0.482 ~ 0.565	0.404 ~ 0.489	N/A	N/A	N/A
Formula to assess RBC count	−57.21 + 0.58 x ED	−67.79 + 0.68 x ED	−46.94 + 0.48 x ED	N/A	N/A	N/A

Note— NECT, non-enhanced CT; ED, electron density image; HU, CT attenuation; Hb, hemoglobin; Hct, hematocrit; RBC, red blood cell; CECT, contrast-enhanced CT; Spearman’s rank correlation coefficient, *r*_*s*_

The mean HU associated with NECT similarly demonstrated positive correlations with Hb, Hct, and RBC count for both males and females. The correlation coefficients for males were 0.801, 0.790, and 0.747, and for females, they were 0.784, 0.783, and 0.716, respectively (all p < 0.001). However, the mean HU achieved with CECT did not correlate with any of these laboratory parameters.

S4 Table summarizes the correlations between blood parameters and cardiac CT values at multiple anatomical sites in CECT. Across all sites, ED values showed consistent moderate positive correlations with hematologic parameters. For Hb, correlation coefficients ranged from *r*_*s*_ = 0.362 to *r*_*s*_ = 0.487 (all p < 0.001). The left ventricular ROI showed the strongest correlation (*r*_*s*_ = 0.487, p < 0.001), followed by the ascending and descending aorta (*r*_*s*_ = 0.459 and *r*_*s*_ = 0.457, respectively; both p < 0.001). Correlations were lower in the pulmonary trunk and right ventricle (*r*_*s*_ = 0.366 and *r*_*s*_ = 0.362, respectively) but remained statistically significant. By contrast, HU values showed weak or non-significant correlations across all anatomical sites, regardless of chamber selection.

In the sensitivity analysis stratified by CT-to-laboratory interval, Spearman’s rank correlation coefficients between mean ED and hematologic parameters remained consistent across all temporal subgroups in both CECT and NECT cohorts (S5 Table). For CECT, correlations between mean ED and Hb were *r*_*s*_ = 0.512 and *r*_*s*_ = 0.508 for the < 24-hour and <48-hour subgroups, respectively, compared with *r*_*s*_ = 0.556 in the full cohort. Similar trends were observed for Hct and RBC count. For NECT, correlations between mean ED and Hb were *r*_*s*_ = 0.724 and *r*_*s*_ = 0.724 for the < 24-hour and <48-hour subgroups, respectively, compared with *r*_*s*_ = 0.756 in the full cohort, with comparable consistency observed for Hct and RBC count. In contrast, mean HU in CECT showed consistently negligible or weakly negative correlations with all hematologic parameters across all temporal subgroups, consistent with the primary analysis.

To adjust for potential demographic confounding, partial correlation analysis controlling for age and sex was performed. After adjustment, partial correlation coefficients between mean ED and Hb remained substantial in both NECT (*r*_*s*_ = 0.694, R² = 0.482) and CECT (*r*_*s*_ = 0.509, R² = 0.259), and were consistent across all CT-to-laboratory interval subgroups (S6 Table). These findings indicate that the associations between mean ED and hematologic parameters are not attributable to demographic confounding.

In addition, bootstrap internal validation (1,000 resamples) of the age- and sex-adjusted partial correlation coefficients demonstrated negligible bias and narrow 95% confidence intervals for all mean ED estimates in both CECT and NECT, confirming the stability of the observed associations (S7 Table). By contrast, the bootstrap 95% confidence interval (CI) for mean HU in CECT crossed zero for RBC count (95% CI −0.025–0.054), further confirming the absence of a clinically meaningful association between HU and hematologic parameters in the contrast-enhanced setting.

### Differentiation between anemia severity groups based on CT measurement

The results of the ROC analysis are presented in Table 4 and S2 Figure. The areas under the ROC curves (AUCs) for distinguishing anemia and severe anemia using ED on NECT were 0.855 (95% CI, 0.834–0.876) in males and 0.825 (95% CI, 0.803–0.847) in females for anemia, and 0.933 (95% CI, 0.908–0.958) in males and 0.956 (95% CI, 0.941–0.972) in females for severe anemia. The AUCs for distinguishing anemia and severe anemia using ED on CECT were 0.738 (95% CI, 0.711–0.766) in males and 0.727 (95% CI, 0.699–0.754) in females for anemia, and 0.850 (95% CI, 0.801–0.898) in males and 0.895 (95% CI, 0.866–0.925) in females for severe anemia.

**Table 4:** Receiver operating characteristic curve analysis of CT measurements as discriminator of anemia or severe anemia.

NECT cohort	Mean ED			Mean HU		
	All patients	Male	Female	All patients	Male	Female
***Anemia vs Normal***						
AUC	0.808	0.855	0.825	0.842	0.875	0.848
95% confidence interval	0.792-0.824	0.834-0.876	0.803-0.847	0.827-0.857	0.856-0.894	0.827-0.869
Cutoff value	104.81	105.01	104.81	42.65	44.53	42.07
Sensitivity (%)	68.67	77.78	78.47	65.47	75.76	66.78
Specificity (%)	78.04	77.45	69.93	86.97	82.12	87.45
Youden index J	0.467	0.552	0.484	0.524	0.579	0.542
***Severe anemia vs others***						
AUC	0.949	0.933	0.956	0.958	0.928	0.971
95% confidence interval	0.940-0.962	0.908-0.958	0.941-0.972	0.940-0.976	0.882-0.975	0.957-0.986
Cutoff value	104.39	104.51	104.27	37.78	39.93	37.61
Sensitivity (%)	92.41	93.48	92.93	88.97	95.65	91.92
Specificity (%)	86.12	82.63	88.77	92.00	81.92	93.95
Youden index J	0.785	0.761	0.817	0.810	0.776	0.859
**CECT cohort**	**Mean ED**			**Mean HU**		
	**All patients**	**Male**	**Female**	**All patients**	**Male**	**Female**
***Anemia vs Normal***						
AUC	0.724	0.738	0.727	0.515	0.531	0.516
95% confidence interval	0.705-0.744	0.711-0.766	0.699-0.754	0.492-0.537	0.498-0.564	0.484-0.547
Cutoff value	106.25	106.55	106.25	224.70	250.41	238.84
Sensitivity (%)	53.59	70.10	56.32	23.79	49.64	26.31
Specificity (%)	80.52	66.00	78.55	81.00	56.86	81.18
Youden index J	0.341	0.361	0.349	0.048	0.065	0.0749
***Severe anemia vs others***						
AUC	0.881	0.850	0.895	0.583	0.540	0.632
95% confidence interval	0.856-0.907	0.801-0.898	0.866-0.925	0.534-0.631	0.454-0.626	0.573-0.691
Cutoff value	105.87	106.21	105.87	238.49	232.87	238.34
Sensitivity (%)	77.53	89.66	83.33	48.31	50.00	45.00
Specificity (%)	85.26	69.14	85.89	70.38	65.26	80.45
Youden index J	0.628	0.588	0.692	0.187	0.153	0.255

Note— NECT, non-enhanced CT; ED, electron density image; HU, CT attenuation; AUC, area under the receiver operating characteristic curve; ROC, receiver operator characteristic curve; CECT, contrast-enhanced CT

The cutoff ED values for distinguishing anemia and severe anemia on NECT were 105.01 (sensitivity, 77.78%; specificity, 77.45%) in males and 104.81 (sensitivity, 78.47%; specificity, 69.93%) in females for anemia, and 104.51 (sensitivity, 93.48%; specificity, 82.63%) in males and 104.27 (sensitivity, 92.93%; specificity, 88.77%) in females for severe anemia. The cutoff ED values for distinguishing anemia and severe anemia on CECT were 106.55 (sensitivity, 70.10%; specificity, 66.00%) in males and 106.25 (sensitivity, 56.32%; specificity, 78.55%) in females for anemia, and 106.21 (sensitivity, 89.66%; specificity, 69.14%) in males and 105.87 (sensitivity, 83.33%; specificity, 85.89%) in females for severe anemia.

The AUCs for distinguishing anemia and severe anemia using HU on NECT were 0.875 (95% CI, 0.856–0.894) in males and 0.848 (95% CI, 0.827–0.869) in females for anemia, and 0.928 (95% CI, 0.882–0.975) in males and 0.971 (95% CI, 0.957–0.986) in females for severe anemia. The cutoff HU values for distinguishing anemia and severe anemia on NECT were 44.53 (sensitivity, 75.76%; specificity, 82.12%) in males and 42.07 (sensitivity, 66.78%; specificity, 87.45%) in females for anemia, and 39.93 (sensitivity, 95.65%; specificity, 81.92%) in males and 37.61 (sensitivity, 91.92%; specificity, 93.95%) in females for severe anemia.

However, HU values from CECT performed poorly in differentiating between subjects without anemia and those with any type of anemia, as well as between severe anemia and other conditions, with AUCs ranging from 0.516 to 0.632.

## Discussion

This study demonstrated that, with both NECT and CECT, the mean ED significantly decreased with increasing anemia severity in both males and females, with notable variation among the different anemia severity groups. However, the mean HU achieved with CECT did not exhibit any decreasing trends with increasing anemia severity. Additionally, the mean ED showed strong positive correlations with Hb, Hct, and RBC count with both NECT and CECT, whereas the mean HU correlated with these laboratory parameters only in association with NECT. Differentiating between subjects without anemia and those with any type of anemia, as well as between severe anemia and other conditions using ED with both NECT and CECT, yielded AUCs ranging from 0.724 to 0.895. In contrast, HU values from CECT performed poorly in these differentiation tasks. Despite ongoing efforts to evaluate anemia using CT, the utility of the ED map derived from DECT in the assessment of anemia has not been clearly established. The only recent *in vitro* study reported a positive correlation between ED and Hb/Hct using blood samples from 348 males and 465 females [15]. Their research identified significant differences in ED between blood samples with and without anemia for both sexes, showing AUCs ranging from 0.7 to 0.95, which aligns closely with our findings. However, their study was conducted solely using blood samples, and they did not investigate the role of ED maps in evaluating anemia using CECT. Our study identified significantly decreased ED values in CECT corresponding to the severity of anemia and observed positive correlations between ED and Hb, Hct, and RBC counts across a total of 2,545 subjects, which represents the largest cohort to our knowledge. Additionally, we established ED cutoff values to distinguish between normal subjects and those with anemia, as well as between severe anemia and other conditions.

ED can depict atoms, chemical bonds, and tissue composition, indicating their potential to elucidate cellularity [16,17]. ED quantifies the likelihood of electron presence at specific locations, influenced by the molecular structure of tissues. Phantom studies have demonstrated a direct correlation between tissue ED and physical density [18], providing valuable insights into changes in tissue elemental composition in the context of lesions. Previous research has noted that RBCs mainly consist of solid blood components, with Hct indicating the percentage of these cellular components [15]. Therefore, the positive correlations between ED and these laboratory parameters observed in our study are consistent with the established composition of RBCs and their role in anemia.

Additionally, ED has been reported to be comparable to attenuation in single-energy CT [19,20]. Recent studies have suggested that ED values show no significant correlation with iodine concentration, implying that ED may reflect intrinsic tissue characteristics with relative robustness to contrast enhancement [21]. However, our findings refine this interpretation. Although ED primarily reflects intrinsic tissue composition, our data indicate that iodinated contrast agents contribute additively to measured ED values. This effect is reflected in the systematic upward shift in mean ED in CECT compared with NECT. ED should therefore not be interpreted as completely independent of contrast enhancement. Rather, the diagnostic advantage of ED in CECT appears to arise from the relatively low variance of the iodine-related contribution. Although iodine increases absolute ED values, its variability appears sufficiently constrained to preserve the underlying anemia-related signal. This interpretation is supported by subanalysis across cardiac anatomical sites. Although correlations were lower in the pulmonary trunk and right ventricle than in the aorta and left ventricle, ED values maintained moderate correlations with hematologic parameters across all anatomic sites in CECT. In contrast, HU measurements in CECT are strongly influenced by variability in iodine concentration within the blood pool. This variability overwhelms attenuation differences attributable to Hb concentration and eliminates meaningful correlation with anemia severity. This distinction likely explains why ED, but not HU, retained diagnostic utility in CECT.

Several studies have shown that VNC images from DECT or photon-counting CT can be used to detect and quantify anemia using CECT scans [12–14,22]. These findings, including ours, are clinically significant for oncology patients, who are particularly susceptible to anemia associated with treatments like chemotherapy or conditions such as cancer-associated bleeding. Given that cancer patients frequently undergo CECT scans for diagnostic or evaluative purposes, the ability to predict serum Hb, Hct, and RBC counts from routine CECT scans and perform early anemia evaluation could greatly aid in the timely detection and management of these complications. Consequently, CT imaging could become an even more valuable diagnostic tool in oncology practice.

However, it is important to acknowledge that the AUCs for detecting any-grade anemia on CECT (0.727 in males; 0.738 in females) represent modest discriminatory performance, particularly given that mild anemia constitutes the majority of detected cases. At these operating points, sensitivities of 56–70% and specificities of 66–79% are unlikely to be clinically actionable as stand-alone screening evidence. The primary utility of ED in CECT is therefore best conceptualized as opportunistic detection — the recognition of unexpected or pre-existing anemia in patients already undergoing DECT for an unrelated clinical indication, such as oncology staging, pulmonary embolism workup, or trauma evaluation. This is particularly relevant for severe anemia, for which AUCs exceeded 0.850–0.895 and sensitivities exceeded 77%, representing a clinically meaningful level of discriminatory performance. In this opportunistic context, ED measurement requires no additional scan time, radiation exposure, or contrast administration, as the spectral data are inherently available from the standard acquisition. Automated flagging of reduced blood pool ED values within structured CT report templates or artificial intelligence-based post-processing workflows could prompt clinicians to order confirmatory laboratory testing at an earlier stage than they otherwise might, particularly in oncology patients at elevated risk for treatment-related anemia. Mild anemia detection using ED on CECT should be interpreted as supplementary to, not a replacement for, laboratory testing, and laboratory confirmation remains essential before any clinical decision-making. The feasibility and clinical impact of integrating ED-based anemia flagging into routine imaging workflows warrant prospective evaluation in future studies.

Our study also highlighted the utility of HU in assessing anemia using NECT. In the largest cohort reported in the literature, we established that HU could predict anemia severity and found correlations between HU and Hb, Hct, and RBC count, consistent with previous research [4,7,9,11,23,24]. However, we did not observe any trends or correlations between HU and anemia severity using CECT.

In the present study, a fixed 30-second scan delay was used for all contrast-enhanced CT examinations. Cardiac output is a major determinant of contrast bolus kinetics. High-output states—commonly observed in severe anemia as a compensatory mechanism—can accelerate contrast arrival and washout, whereas low-output states, such as heart failure, may delay peak aortic enhancement [25,26]. Consequently, at a uniform 30-second acquisition time, intravascular iodine concentration—and therefore its contribution to measured blood pool ED—may vary systematically according to individual hemodynamic status. In patients with severe anemia and compensatory hyperdynamic circulation, more rapid bolus transit may reduce aortic iodine concentration at the time of imaging [27], thereby directionally reinforcing ED reductions attributable to decreased RBC mass and potentially strengthening the observed associations. In contrast, in patients with reduced cardiac output, delayed peak enhancement may partially offset the expected ED decrease despite the presence of clinically significant anemia. Because cardiac output and related hemodynamic parameters were not available in this retrospective cohort, adjustment for this potential confounder was not feasible.

Nevertheless, several considerations mitigate the potential influence of this hemodynamic confounding. First, the moderate correlations observed between ED and laboratory parameters in the NECT cohort (*r*_*s*_ = 0.657–0.770). This finding provides independent evidence that ED intrinsically reflects RBC composition and supports the interpretation that the associations observed in the CECT cohort are not merely artifactual. Second, the large size of the CECT cohort (n = 2,545) includes patients with a wide range of physiological and cardiovascular conditions, encompassing both normal and impaired cardiac output states. In a population of this magnitude, inter-individual hemodynamic variability is more likely to contribute to stochastic variability rather than directional bias, thereby tending to dilute, rather than spuriously enhance, the strength of observed correlations. Third, the advantage of ED over conventional HU measurements in the contrast-enhanced setting is highlighted by the near absence of correlation between HU and laboratory parameters. This finding confirms that iodine predominantly determines HU values after contrast administration, rendering HU diagnostically unreliable for anemia detection. In contrast, ED maintained statistically significant and clinically meaningful correlations with Hb, Hct, and RBC count under identical acquisition conditions. The persistence of these associations despite shared exposure to contrast timing and potential hemodynamic variability suggests that ED retains partial functional independence from intravascular iodine concentration, consistent with its underlying physical basis in ED rather than HU. Future prospective studies incorporating bolus-tracking or individualized acquisition timing, along with documentation of hemodynamic variables, would help disentangle iodine-related variability from the intrinsic ED signal associated with RBC composition.

Our study had several limitations. First, this study was conducted at a single institution using a single DECT platform (Philips IQon dual-layer spectral detector CT), which limits the generalizability of our findings. ED quantification methods and calibration standards vary across DECT technologies, and the diagnostic cutoff values identified in this study are therefore platform-dependent and cannot be directly transferred to other scanner types without cross-calibration and external validation. Readers are cautioned against overgeneralizing the reported cutoff values beyond the specific acquisition and reconstruction conditions of this study. Future multicenter studies incorporating diverse DECT platforms—including dual-source and rapid kVp-switching systems—are warranted to establish vendor-neutral ED thresholds and to confirm the generalizability of the observed associations across different imaging environments... Second, our institutional protocol resulted in different radiation doses between NECT and CECT. In addition, information on clinical factors such as body mass index (BMI), which could affect image quality, was unavailable. The extent to which ED values are influenced by radiation dose or image noise remains unclear, warranting further investigation. Third, the 7-day window between CT acquisition and laboratory testing may introduce temporal bias, particularly in hospitalized patients in whom Hb levels can change rapidly. However, a pre-specified sensitivity analysis stratified by CT-to-laboratory interval demonstrated that correlation coefficients between mean ED and hematologic parameters were consistent across all subgroups for both CECT and NECT, supporting the robustness of the primary findings to this inclusion criterion. Fourth, additional unmeasured confounders that may influence blood pool ED and HU measurements include hydration status, renal function, BMI and body habitus, and contrast timing variability arising from differences in cardiac output and venous access. Because these variables were not consistently available in our retrospective dataset, their independent contributions to measured ED values could not be quantified. Future prospective studies should incorporate these variables into a multivariate framework to better isolate the contribution of hematologic parameters to blood pool ED, and to determine whether adjustment for hemodynamic and physiological confounders meaningfully alters the observed associations.

In conclusion, ED derived from DECT showed strong diagnostic utility for anemia detection on NECT and retained meaningful correlations with hematologic parameters on CECT, though its discriminatory performance for any-grade anemia on CECT was modest and should be interpreted as opportunistic detection rather than a stand-alone diagnostic tool. HU on NECT similarly reflected anemia severity, whereas HU on CECT showed no meaningful correlation with hematologic parameters.

## Supporting information

S1 Table Protocols of non-enhanced CT and contrast-enhanced CT. (DOCX)

S2 Table Comparisons of ED at each ROI according to anemia severity in non-enhanced CT and contrast-enhanced CT cohorts. (DOCX)

S3 Table Comparisons of HU at each ROI according to anemia severity in non-enhanced CT and contrast-enhanced CT cohorts. (DOCX)

S4 Table Spearman’s rank correlations between hematologic parameters and cardiac CT values by anatomical location in CECT. (DOCX)

S5 Table Sensitivity analysis: Spearman’s rank correlation coefficients (*r*_*s*_) between CT measurements and hematologic parameters stratified by CT-to-laboratory interval. (DOCX)

S6 Table Partial correlation coefficients (*r*_*s*_) and coefficients of determination (R²) between mean ED/ mean HU and hematologic parameters, adjusted for age and sex, stratified by CT-to-laboratory interval. (DOCX)

S7 Table Bootstrap internal validation (1,000 resamples) of partial correlation coefficients (r_s_) between mean ED/ mean HU and hematologic parameters, adjusted for age and sex. (DOCX)

S1 Fig Bland–Altman plots demonstrating agreement between laboratory-measured hemoglobin, hematocrit, and red blood cell counts and electron density (ED)–derived predicted values. (A) Contrast-enhanced CT. (B) Non-enhanced CT.
(DOCX)

S2 Fig Receiver operating characteristic (ROC) curves for differentiating between subjects with and without anemia for both males and females, and between severe anemia and other categories for males and females for non-enhanced and contrast-enhanced CT. (DOCX)

S1 File Study dataset. Individual-level age data were removed from the supplementary dataset on privacy grounds; readers wishing to replicate age-stratified subanalyses should be aware of this constraint.
(XLSX)

## Abbreviations

ED: Electron density images derived from dual-energy CT
DECT: Dual-energy CT
NECT: Non-enhanced CT
CECT: Contrast-enhanced CT
Hb: Hemoglobin
Hct: Hematocrit
RBC: Red blood cell
