# Supplementary material for: Evaluation of anemia in non-enhanced and contrast-enhanced dual-energy CT using electron density imaging
Source: PLoS One. 2026 Jul 2;21(7):e0352504. doi: 10.1371/journal.pone.0352504 (PMC13327118; doi:10.1371/journal.pone.0352504)
Supplement: S1 Table — (DOCX) [file pone.0352504.s001.docx]

**S1 Table**. Protocols of non-enhanced CT and contrast-enhanced CT.

|  | **Non-enhanced CT** | **Contrast-enhanced CT** |
| --- | --- | --- |
| kVp | 120 | 120 |
| Dose Right Index (DRI) | 16 | 20 |
| Reference mAs | 66 | 103 |
| Collimation | 64 × 0.625 mm | 64 × 0.625 mm |
| Slice thickness, mm | 3 | 3 |
| Beam width, mm | 40 | 40 |
| Focal spot resolution | High | High |
| Pitch | 0.609 | 0.609 |
| Rotation time | 0.4 | 0.4 |
| Dose modulation | Z modulation 3D modulation | Z modulation 3D modulation |
