# Supplementary material for: Evaluation of anemia in non-enhanced and contrast-enhanced dual-energy CT using electron density imaging
Source: PLoS One. 2026 Jul 2;21(7):e0352504. doi: 10.1371/journal.pone.0352504 (PMC13327118; doi:10.1371/journal.pone.0352504)
Supplement: S2 Table — (DOCX) [file pone.0352504.s002.docx]

**S2 Table**. Comparisons of ED at each ROI according to anemia severity in non-enhanced CT and contrast-enhanced CT cohorts.

| **Non-enhanced CT cohort** | **No anemia** | **Mild anemia** | **Moderate anemia** | **Severe anemia** | ***p*-value** |
| --- | --- | --- | --- | --- | --- |
| *All patients* |  |  |  |  |  |
| ED of ascending aorta | 104.98±0.43 | 104.62±0.44* | 104.07±0.49*† | 103.65±0.54*†§ | <0.001 |
| ED of pulmonary trunk | 105.07±0.47 | 104.69±0.48* | 104.11±0.53*† | 103.73±0.55*†§ | <0.001 |
| ED of descending aorta | 105.24±0.47 | 104.83±0.53* | 104.13±0.63*† | 103.79±0.68*†§ | <0.001 |
| ED of right ventricle | 105.01±0.45 | 104.67±0.57* | 104.16±0.54*† | 103.73±0.63*†§ | <0.001 |
| ED of left ventricle | 105.01±0.47 | 104.71±0.49* | 104.19±0.63*† | 103.83±0.55*†§ | <0.001 |
| *Male* |  |  |  |  |  |
| ED of ascending aorta | 105.16±0.41 | 104.70±0.44* | 104.05±0.47*† | 103.83±0.52*†§ | <0.001 |
| ED of pulmonary trunk | 105.26±0.45 | 104.76±0.48* | 104.12±0.48*† | 103.97±0.53*† | <0.001 |
| ED of descending aorta | 105.42±0.43 | 104.89±0.55* | 104.10±0.59*† | 104.04±0.48*† | <0.001 |
| ED of right ventricle | 105.19±0.44 | 104.74±0.48* | 104.20±0.51*† | 103.87±0.55*†§ | <0.001 |
| ED of left ventricle | 105.20±0.46 | 104.78±0.51* | 104.29±0.56*† | 103.98±0.58*†§ | <0.001 |
| *Female* |  |  |  |  |  |
| ED of ascending aorta | 104.86±0.40 | 104.51±0.42* | 104.08±0.50*† | 103.56±0.54*†§ | <0.001 |
| ED of pulmonary trunk | 104.94±0.44 | 104.59±0.46* | 104.10±0.58*† | 103.61±0.53*†§ | <0.001 |
| ED of descending aorta | 105.13±0.47 | 104.75±0.50* | 104.17±0.67*† | 103.67±0.72*†§ | <0.001 |
| ED of right ventricle | 104.89±0.42 | 104.56±0.44* | 104.12±0.56*† | 103.66±0.66*†§ | <0.001 |
| ED of left ventricle | 104.87±0.42 | 104.60±0.44* | 104.08±0.68*† | 103.76±0.52*†§ | <0.001 |
| **Contrast-enhanced CT cohort** | **No anemia** | **Mild anemia** | **Moderate anemia** | **Severe anemia** | ***p*-value** |
| *All patients* |  |  |  |  |  |
| ED of ascending aorta | 106.65±0.58 | 106.35±0.62* | 105.77±0.74*† | 105.43±0.71*†§ | <0.001 |
| ED of pulmonary trunk | 106.61±0.74 | 106.33±0.78* | 105.67±0.84*† | 105.45±0.83*† | <0.001 |
| ED of descending aorta | 106.98±0.62 | 106.59±0.65* | 105.90±0.83*† | 105.73±0.81*† | <0.001 |
| ED of right ventricle | 106.46±0.74 | 106.20±0.79* | 105.64±0.80*† | 105.35±0.84*†§ | <0.001 |
| ED of left ventricle | 106.62±0.61 | 106.30±0.60* | 105.79±0.72*† | 105.33±0.65*†§ | <0.001 |
| *Male* |  |  |  |  |  |
| ED of ascending aorta | 106.75±0.56 | 106.40±0.61* | 105.68±0.61*† | 105.48±0.75*† | <0.001 |
| ED of pulmonary trunk | 106.69±0.75 | 106.35±0.75* | 105.65±0.63*† | 105.65±0.86*† | <0.001 |
| ED of descending aorta | 107.01±0.61 | 106.58±0.66* | 105.84±0.71*† | 105.84±0.79*† | <0.001 |
| ED of right ventricle | 106.57±0.75 | 106.22±0.78* | 105.56±0.63*† | 105.49±0.74*† | <0.001 |
| ED of left ventricle | 106.73±0.62 | 106.34±0.61* | 105.71±0.65*† | 105.50±0.60*† | <0.001 |
| *Female* |  |  |  |  |  |
| ED of ascending aorta | 106.59±0.58 | 106.28±0.64* | 105.87±0.85*† | 105.41±0.69*†§ | <0.001 |
| ED of pulmonary trunk | 106.57±0.73 | 106.29±0.81* | 105.69±1.02*† | 105.36±0.79*†§ | <0.001 |
| ED of descending aorta | 106.97±0.64 | 106.61±0.64* | 105.97±0.94*† | 105.68±0.82*†§ | <0.001 |
| ED of right ventricle | 106.39±0.73 | 106.18±0.81* | 105.72±0.95*† | 105.29±0.88*†§ | <0.001 |
| ED of left ventricle | 106.54±0.59 | 106.23±0.58* | 105.88±0.79*† | 105.25±0.66*†§ | <0.001 |

**p*<0.05 (vs. No anemia)

†*p*<0.05 (vs. Mild anemia)

§*p*<0.05 (vs. Moderate anemia)
