# Supplementary material for: Evaluation of anemia in non-enhanced and contrast-enhanced dual-energy CT using electron density imaging
Source: PLoS One. 2026 Jul 2;21(7):e0352504. doi: 10.1371/journal.pone.0352504 (PMC13327118; doi:10.1371/journal.pone.0352504)
Supplement: S3 Table — (DOCX) [file pone.0352504.s003.docx]

**S3 Table**. Comparisons of HU at each ROI according to anemia severity in non-enhanced CT and contrast-enhanced CT cohorts.

| **Non-enhanced CT cohort** | **No anemia** | **Mild anemia** | **Moderate anemia** | **Severe anemia** | ***p*-value** |
| --- | --- | --- | --- | --- | --- |
| *All patients* |  |  |  |  |  |
| HU of ascending aorta | 45.04±4.12 | 41.09±4.29* | 36.30±4.69*† | 30.96±6.42*†§ | <0.001 |
| HU of pulmonary trunk | 46.16±4.69 | 42.34±4.61* | 37.29±4.90*† | 32.33±6.89*†§ | <0.001 |
| HU of descending aorta | 48.87±5.30 | 44.47±6.09* | 38.12±7.12*† | 32.31±8.67*†§ | <0.001 |
| HU of right ventricle | 44.55±4.54 | 41.16±4.83* | 36.80±5.58*† | 31.10±7.84*†§ | <0.001 |
| HU of left ventricle | 44.28±4.91 | 41.31±5.40* | 36.58±6.55*† | 31.22±7.18*†§ | <0.001 |
| *Male* |  |  |  |  |  |
| HU of ascending aorta | 46.24±3.85 | 41.44±4.46* | 35.38±4.74*† | 32.57±6.99*†§ | <0.001 |
| HU of pulmonary trunk | 47.39±4.75 | 42.63±4.83* | 37.00±4.87*† | 35.20±5.55*† | <0.001 |
| HU of descending aorta | 50.13±5.34 | 44.64±6.32* | 37.42±6.41*† | 35.98±6.76*† | <0.001 |
| HU of right ventricle | 45.77±4.48 | 41.28±5.14* | 36.97±5.83*† | 32.19±7.66*†§ | <0.001 |
| HU of left ventricle | 45.72±4.88 | 41.58±5.67* | 37.69±6.59*† | 32.73±7.54*†§ | <0.001 |
| *Female* |  |  |  |  |  |
| HU of ascending aorta | 44.25±4.10 | 40.61±3.99* | 37.27±4.45*† | 30.21±6.03*†§ | <0.001 |
| HU of pulmonary trunk | 45.35±4.48 | 41.94±4.28* | 37.60±4.93*† | 31.00±7.07*†§ | <0.001 |
| HU of descending aorta | 48.02±5.10 | 44.24±5.77* | 38.86±7.77*† | 30.61±8.96*†§ | <0.001 |
| HU of right ventricle | 43.74±4.41 | 40.99±4.38* | 36.62±5.33*† | 30.59±7.92*†§ | <0.001 |
| HU of left ventricle | 43.32±4.69 | 40.94±4.99* | 35.39±6.33*† | 30.51±6.94*†§ | <0.001 |
| **Contrast-enhanced CT cohort** | **No anemia** | **Mild anemia** | **Moderate anemia** | **Severe anemia** | ***p*-value** |
| *Male* |  |  |  |  |  |
| HU of ascending aorta | 277.39±49.42 | 276.96±51.29 | 276.70±61.75 | 265.10±62.71*† | 0.03 |
| HU of pulmonary trunk | 261.38±75.03 | 267.57±78.22 | 262.36±83.32 | 263.74±77.55 | 0.311 |
| HU of descending aorta | 277.40±48.71 | 274.83±60.36 | 267.83±60.36 | 260.16±61.64*† | <0.011 |
| HU of right ventricle | 251.08±75.21 | 256.65±79.24 | 250.04±82.94 | 246.43±74.98 | 0.222 |
| HU of left ventricle | 259.86±49.01 | 258.10±50.72 | 254.65±57.05 | 232.62±59.08*†§ | <0.001 |
| *Female* |  |  |  |  |  |
| HU of ascending aorta | 262.79±44.89 | 269.67±49.26 | 264.74±53.34 | 260.07±56.17 | 0.096 |
| HU of pulmonary trunk | 246.02±74.11 | 258.17±78.58 | 246.99±75.66 | 263.88±86.98 | 0.042 |
| HU of descending aorta | 261.16±44.38 | 265.72±47.36 | 254.50±51.90 | 251.94±53.81 | 0.039 |
| HU of right ventricle | 238.52±78.26 | 248.22±79.85 | 234.30±72.54 | 253.50±77.75 | 0.109 |
| HU of left ventricle | 244.97±45.39 | 249.90±50.47 | 242.68±54.21 | 236.34±53.96 | 0.105 |
| *Women* |  |  |  |  |  |
| HU of ascending aorta | 287.05±49.94 | 286.96±52.39 | 290.29±67.93 | 267.53±65.73*†§ | 0.002 |
| HU of pulmonary trunk | 271.54±73.94 | 280.46±75.95 | 279.84±88.57 | 263.38±72.95 | 0.092 |
| HU of descending aorta | 288.16±48.49 | 286.67±50.42 | 282.99±65.86 | 264.13±64.92* | <0.001 |
| HU of right ventricle | 259.39±71.98 | 268.19±77.01 | 267.93±90.58 | 243.02±73.70† | 0.009 |
| HU of left ventricle | 269.71±48.86 | 269.34±48.93 | 268.25±57.49 | 230.82±61.53*†§ | <0.001 |

**p*<0.05 (vs. No anemia)

†*p*<0.05 (vs. Mild anemia)

§*p*<0.05 (vs. Moderate anemia)
