# Supplementary material for: Evaluation of anemia in non-enhanced and contrast-enhanced dual-energy CT using electron density imaging
Source: PLoS One. 2026 Jul 2;21(7):e0352504. doi: 10.1371/journal.pone.0352504 (PMC13327118; doi:10.1371/journal.pone.0352504)
Supplement: S4 Table — (DOCX) [file pone.0352504.s004.docx]

**S4 Table**. Spearman’s rank correlations between hematologic parameters and cardiac CT values by anatomical location in CECT.

|  | **Ascending aorta** | | **Pulmonary trunk** | | **Descending aorta** | | **Right ventricle** | | **Left ventricle** | |
| --- | --- | --- | --- | --- | --- | --- | --- | --- | --- | --- |
|  | **ED** | **HU** | **ED** | **HU** | **ED** | **HU** | **ED** | **HU** | **ED** | **HU** |
| ***Hb*** |  |  |  |  |  |  |  |  |  |  |
| *r_s_* | 0.459 | −0.029 | 0.366 | −0.077 | 0.457 | −0.005 | 0.362 | −0.059 | 0.487 | <0.001 |
| Significant level | <0.001 | 0.138 | <0.001 | <0.001 | <0.001 | 0.792 | <0.001 | 0.003 | <0.001 | 0.982 |
| ***Hct*** |  |  |  |  |  |  |  |  |  |  |
| *r_s_* | 0.439 | −0.024 | 0.351 | −0.074 | 0.439 | 0.001 | 0.339 | −0.063 | 0.466 | 0.006 |
| Significant level | <0.001 | 0.222 | <0.001 | <0.001 | <0.001 | 0.952 | <0.001 | 0.002 | <0.001 | 0.764 |
| ***RBC count*** |  |  |  |  |  |  |  |  |  |  |
| *r_s_* | 0.400 | −0.058 | 0.307 | −0.103 | 0.403 | −0.027 | 0.297 | −0.090 | 0.420 | −0.026 |
| Significant level | <0.001 | 0.003 | <0.001 | <0.001 | <0.001 | 0.177 | <0.001 | <0.001 | <0.001 | 0.183 |
| Note— CECT, contrast-enhanced CT; ED, electron density image; HU, CT attenuation; Hb, hemoglobin; Hct, hematocrit; RBC, red blood cell | | | | | | | | | | |
