# Supplementary material for: Evaluation of anemia in non-enhanced and contrast-enhanced dual-energy CT using electron density imaging
Source: PLoS One. 2026 Jul 2;21(7):e0352504. doi: 10.1371/journal.pone.0352504 (PMC13327118; doi:10.1371/journal.pone.0352504)
Supplement: S5 Table — (DOCX) [file pone.0352504.s005.docx]

**S5 Table**. Sensitivity analysis: Spearman’s rank correlation coefficients (*r_s_*) between CT measurements and hematologic parameters stratified by CT-to-laboratory interval.

| **NECT** | | |
| --- | --- | --- |
|  | **<24 hours (0-24 hours)** | **<48 hours (0-48 hours)** |
| **n** | **1,692** | **1,896** |
| ***Hb*** | | |
| Mean ED (*r_s_*) | 0.724 | 0.724 |
| Mean HU (*r_s_*) | 0.761 | 0.753 |
| ***Hct*** | | |
| Mean ED (*r_s_*) | 0.697 | 0.695 |
| Mean HU (*r_s_*) | 0.754 | 0.745 |
| ***RBC count*** | | |
| Mean ED (*r_s_*) | 0.651 | 0.65 |
| Mean HU (*r_s_*) | 0.699 | 0.694 |
| **CECT** | | |
|  | **<24 hours (0-24 hours)** | **<48 hours (0-48 hours)** |
| **n** | **1,739** | **1,947** |
| ***Hb*** | | |
| Mean ED (*r_s_*) | 0.512 | 0.508 |
| Mean HU (*r_s_*) | −0.002 | −0.016 |
| ***Hct*** | | |
| Mean ED (*r_s_*) | 0.489 | 0.484 |
| Mean HU (*r_s_*) | 0 | −0.013 |
| ***RBC count*** | | |
| Mean ED (*r_s_*) | 0.443 | 0.438 |
| Mean HU (*r_s_*) | −0.043 | −0.053* |

All correlations were statistically significant (*p* < 0.001) unless otherwise noted. **p* < 0.05 (not significant at *p* < 0.001 threshold). ED, electron density; HU, CT attenuation (Hounsfield unit); Hb, hemoglobin; Hct, hematocrit; RBC, red blood cell; CECT, contrast-enhanced CT; NECT, non-enhanced CT.
