# Supplementary material for: Evaluation of anemia in non-enhanced and contrast-enhanced dual-energy CT using electron density imaging
Source: PLoS One. 2026 Jul 2;21(7):e0352504. doi: 10.1371/journal.pone.0352504 (PMC13327118; doi:10.1371/journal.pone.0352504)
Supplement: S6 Table — (DOCX) [file pone.0352504.s006.docx]

**S6 Table**. Partial correlation coefficients (*r_s_*) and coefficients of determination (R^2^) between mean ED/mean HU and hematologic parameters, adjusted for age and sex, stratified by CT-to-laboratory interval.

| **NECT** | | | | |
| --- | --- | --- | --- | --- |
| **Parameter** | **CT measurements** | **All** | **<24 hours (0-24 hours)** | **<48 hours (0-48 hours)** |
| ***Hb*** | **Mean ED** | *r_s_* = 0.694 | *r_s_* = 0.713 | *r_s_* = 0.709 |
|  |  | R^2^ = 0.482 | R^2^ = 0.508 | R^2^ = 0.502 |
|  | **Mean HU** | *r_s_* = 0.735 | *r_s_* = 0.758 | *r_s_* = 0.749 |
|  |  | R^2^ = 0.540 | R^2^ = 0.575 | R^2^ = 0.561 |
| ***Hct*** | **Mean ED** | *r_s_* = 0.664 | *r_s_* = 0.684 | *r_s_* = 0.678 |
|  |  | R^2^ = 0.441 | R^2^ = 0.468 | R^2^ = 0.460 |
|  | **Mean HU** | *r_s_* = 0.725 | *r_s_* = 0.749 | *r_s_* = 0.738 |
|  |  | R^2^ = 0.526 | R^2^ = 0.561 | R^2^ = 0.545 |
| ***RBC count*** | **Mean ED** | *r_s_* = 0.613 | *r_s_* = 0.635 | *r_s_* = 0.631 |
|  |  | R^2^ = 0.376 | R^2^ = 0.403 | R^2^ = 0.398 |
|  | **Mean HU** | *r_s_* = 0.668 | *r_s_* = 0.688 | *r_s_* = 0.679 |
|  |  | R^2^ = 0.446 | R^2^ = 0.473 | R^2^ = 0.461 |
| **CECT** | | | | |
| **Parameter** | **Metric** | **All** | **<24 hours (0-24 hours)** | **<48 hours (0-48 hours)** |
| ***Hb*** | **Mean ED** | *r_s_* = 0.509 | *r_s_* = 0.532 | *r_s_* = 0.526 |
|  |  | R^2^ = 0.259 | R^2^ = 0.283 | R^2^ = 0.277 |
|  | **Mean HU** | *r_s_* = 0.055 | *r_s_* = 0.080 | *r_s_* = 0.078 |
|  |  | R^2^ = 0.003 | R^2^ = 0.006 | R^2^ = 0.006 |
| ***Hct*** | **Mean ED** | *r_s_* = 0.482 | *r_s_* = 0.507 | *r_s_* = 0.500 |
|  |  | R^2^ = 0.233 | R^2^ = 0.257 | R^2^ = 0.250 |
|  | **Mean HU** | *r_s_* = 0.054 | *r_s_* = 0.079 | *r_s_* = 0.077 |
|  |  | R^2^ = 0.003 | R^2^ = 0.006 | R^2^ = 0.006 |
| ***RBC count*** | **Mean ED** | *r_s_* = 0.435 | *r_s_* = 0.461 | *r_s_* = 0.453 |
|  |  | R^2^ = 0.189 | R^2^ = 0.212 | R^2^ = 0.205 |
|  | **Mean HU** | *r_s_* = 0.017 | *r_s_* = 0.035 | *r_s_* = 0.035 |
|  |  | R^2^ = 0.000 | R^2^ = 0.001 | R^2^ = 0.001 |

All correlations were statistically significant (*p* < 0.001) unless otherwise noted. Partial correlations were adjusted for age and sex. R^2^ was calculated as the square of the partial Spearman’s rank correlation coefficient (*r*_s_^2^), representing the proportion of variance in hematologic parameters explained by the CT measurement after demographic adjustment. ED, electron density; HU, Hounsfield unit; Hb, hemoglobin; Hct, hematocrit; RBC, red blood cell; CECT, contrast-enhanced CT; NECT, non-enhanced CT.
