# Supplementary material for: Evaluation of anemia in non-enhanced and contrast-enhanced dual-energy CT using electron density imaging
Source: PLoS One. 2026 Jul 2;21(7):e0352504. doi: 10.1371/journal.pone.0352504 (PMC13327118; doi:10.1371/journal.pone.0352504)
Supplement: S7 Table — (DOCX) [file pone.0352504.s007.docx]

**S7 Table**. Bootstrap internal validation (1,000 resamples) of partial correlation coefficients (*r_s_*) between mean ED/mean HU and hematologic parameters, adjusted for age and sex.

| **NECT** | | | | | | | |
| --- | --- | --- | --- | --- | --- | --- | --- |
| **Parameter** | **CT measurements** | **Estimate (*r_s_*)** | **Bias** | **SE** | **95% CI Lower** | **95% CI Upper** | **R^2^** |
| ***Hb*** | **Mean ED** | 0.694 | −0.0001 | 0.0117 | 0.671 | 0.717 | 0.482 |
|  | **Mean HU** | 0.735 | −0.0004 | 0.0111 | 0.713 | 0.756 | 0.540 |
| ***Hct*** | **Mean ED** | 0.664 | −0.0002 | 0.0125 | 0.639 | 0.688 | 0.441 |
|  | **Mean HU** | 0.725 | −0.0003 | 0.0113 | 0.702 | 0.748 | 0.526 |
| ***RBC count*** | **Mean ED** | 0.613 | 0.0005 | 0.0135 | 0.587 | 0.641 | 0.376 |
|  | **Mean HU** | 0.668 | 0.0003 | 0.0128 | 0.642 | 0.693 | 0.446 |
| **CECT** | | | | | | | |
| **Parameter** | **CT measurements** | **Estimate (*r_s_*)** | **Bias** | **SE** | **95% CI Lower** | **95% CI Upper** | **R^2^** |
| ***Hb*** | **Mean ED** | 0.509 | −0.0004 | 0.0157 | 0.477 | 0.538 | 0.259 |
|  | **Mean HU** | 0.055 | −0.0007 | 0.0202 | 0.015 | 0.093 | 0.003 |
| ***Hct*** | **Mean ED** | 0.482 | −0.0002 | 0.0163 | 0.450 | 0.513 | 0.233 |
|  | **Mean HU** | 0.054 | −0.0006 | 0.0205 | 0.011 | 0.092 | 0.003 |
| ***RBC count*** | **Mean ED** | 0.435 | −0.0002 | 0.017 | 0.400 | 0.467 | 0.189 |
|  | **Mean HU** | 0.017* | −0.0007 | 0.0204 | **−**0.025***** | 0.054 | 0 |

Bootstrap analysis performed using ordinary nonparametric bootstrap with 1,000 resamples. Partial correlations were adjusted for age and sex. SE, standard error; CI, confidence interval; *R^2^ = r_s_^2^, representing the proportion of variance explained after demographic adjustment.* Values with * indicate 95% CI crossing zero (i.e., non-significant association). ED, electron density; HU, Hounsfield unit; Hb, hemoglobin; Hct, hematocrit; RBC, red blood cell; CECT, contrast-enhanced CT; NECT, non-enhanced CT.
