# Supplementary material for: Evaluation of anemia in non-enhanced and contrast-enhanced dual-energy CT using electron density imaging
Source: PLoS One. 2026 Jul 2;21(7):e0352504. doi: 10.1371/journal.pone.0352504 (PMC13327118; doi:10.1371/journal.pone.0352504)

S1 Figure. Bland–Altman plots demonstrating agreement between laboratory-measured hemoglobin, hematocrit, and red blood cell counts and electron density (ED)-derived predicted values. (A) Contrast-enhanced CT. (B) Non-enhanced CT.

(A)


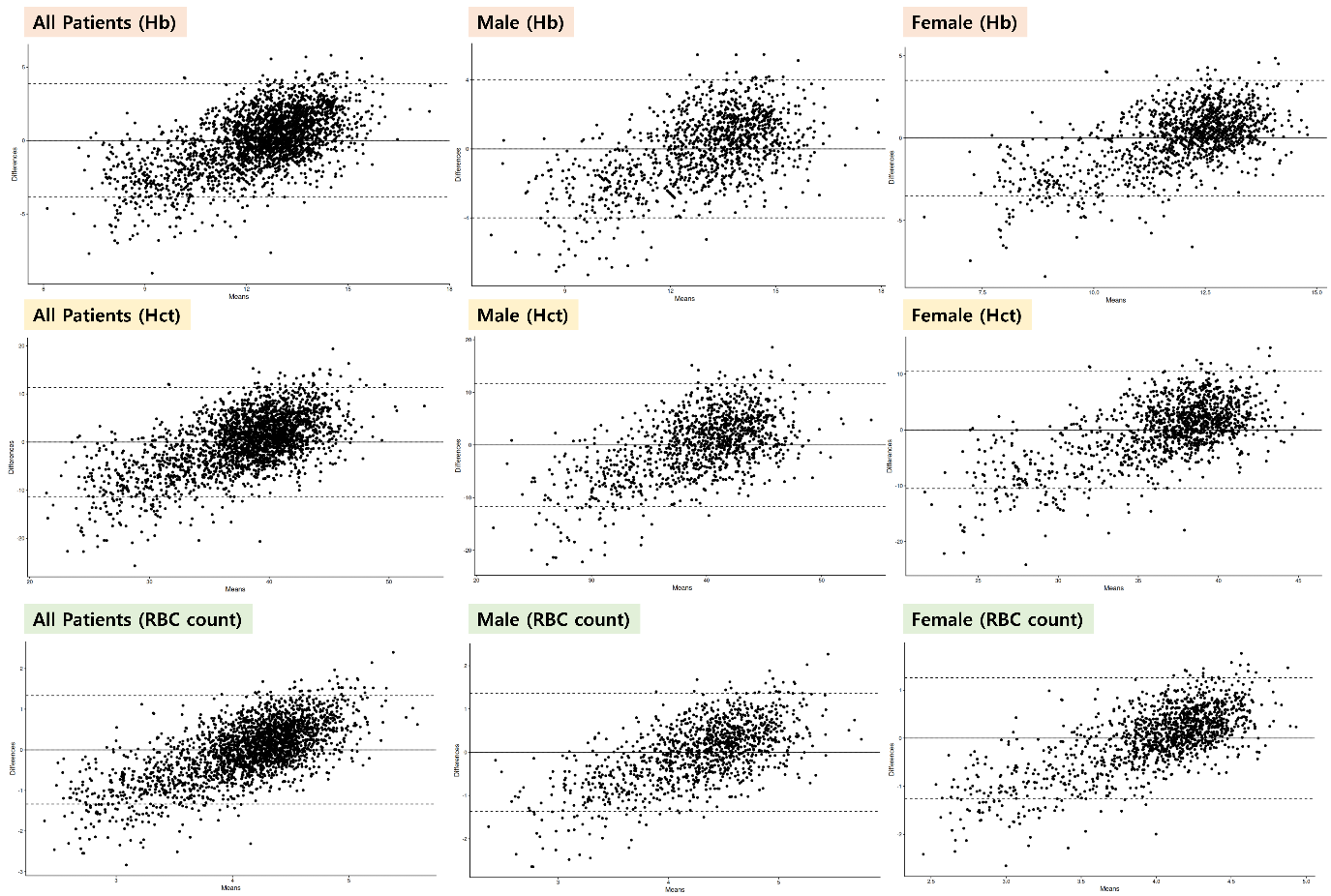


(B)


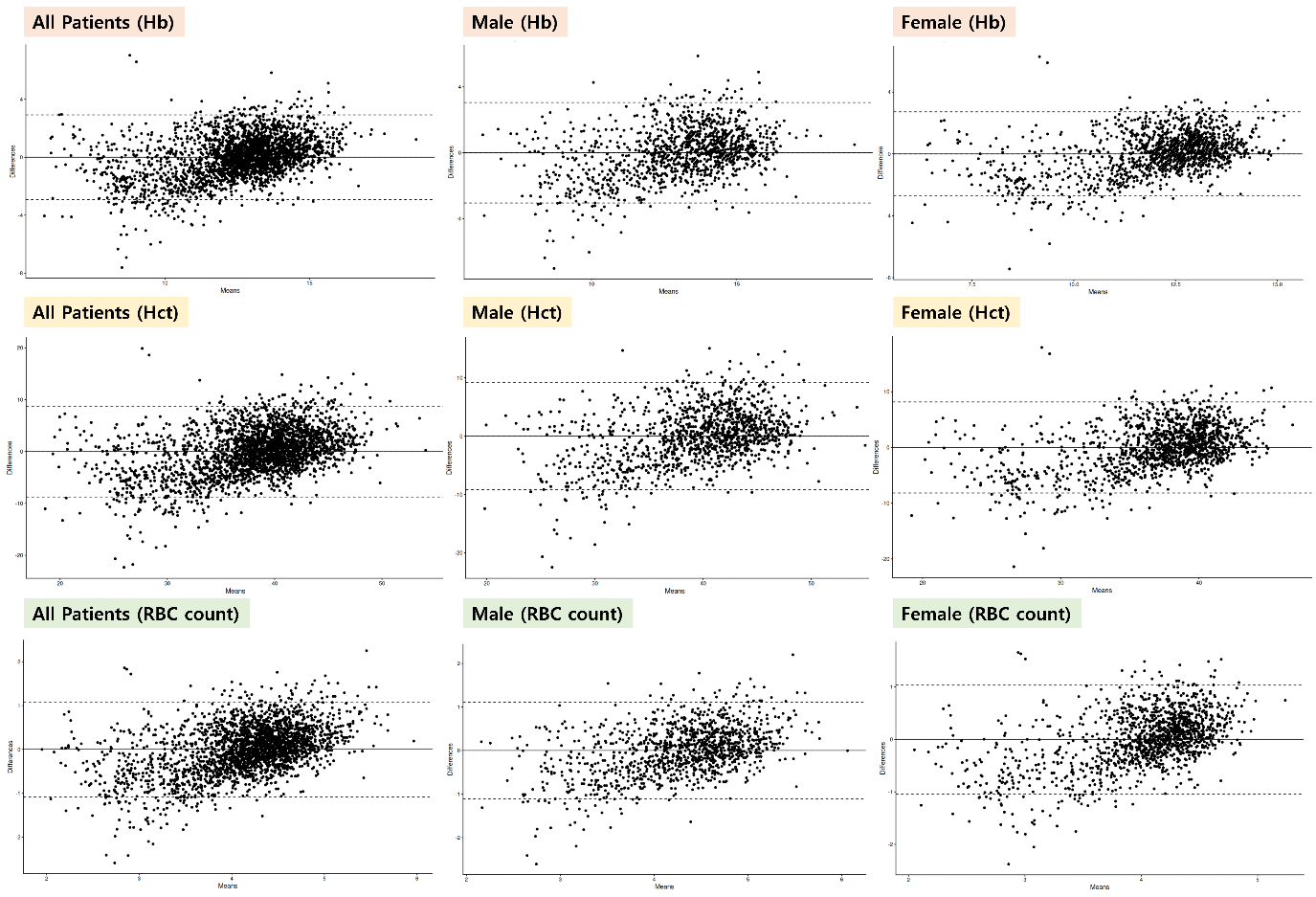

Supplement: S1 Fig — (A) Contrast-enhanced CT. (B) Non-enhanced CT. (DOCX) [file pone.0352504.s008.docx]
