# Supplementary material for: Evaluation of anemia in non-enhanced and contrast-enhanced dual-energy CT using electron density imaging
Source: PLoS One. 2026 Jul 2;21(7):e0352504. doi: 10.1371/journal.pone.0352504 (PMC13327118; doi:10.1371/journal.pone.0352504)

S2 Figure. Receiver operating characteristic (ROC) curves for differentiating between subjects with and without anemia for both males and females, and between severe anemia and other categories for males and females for non-enhanced and contrast-enhanced CT.


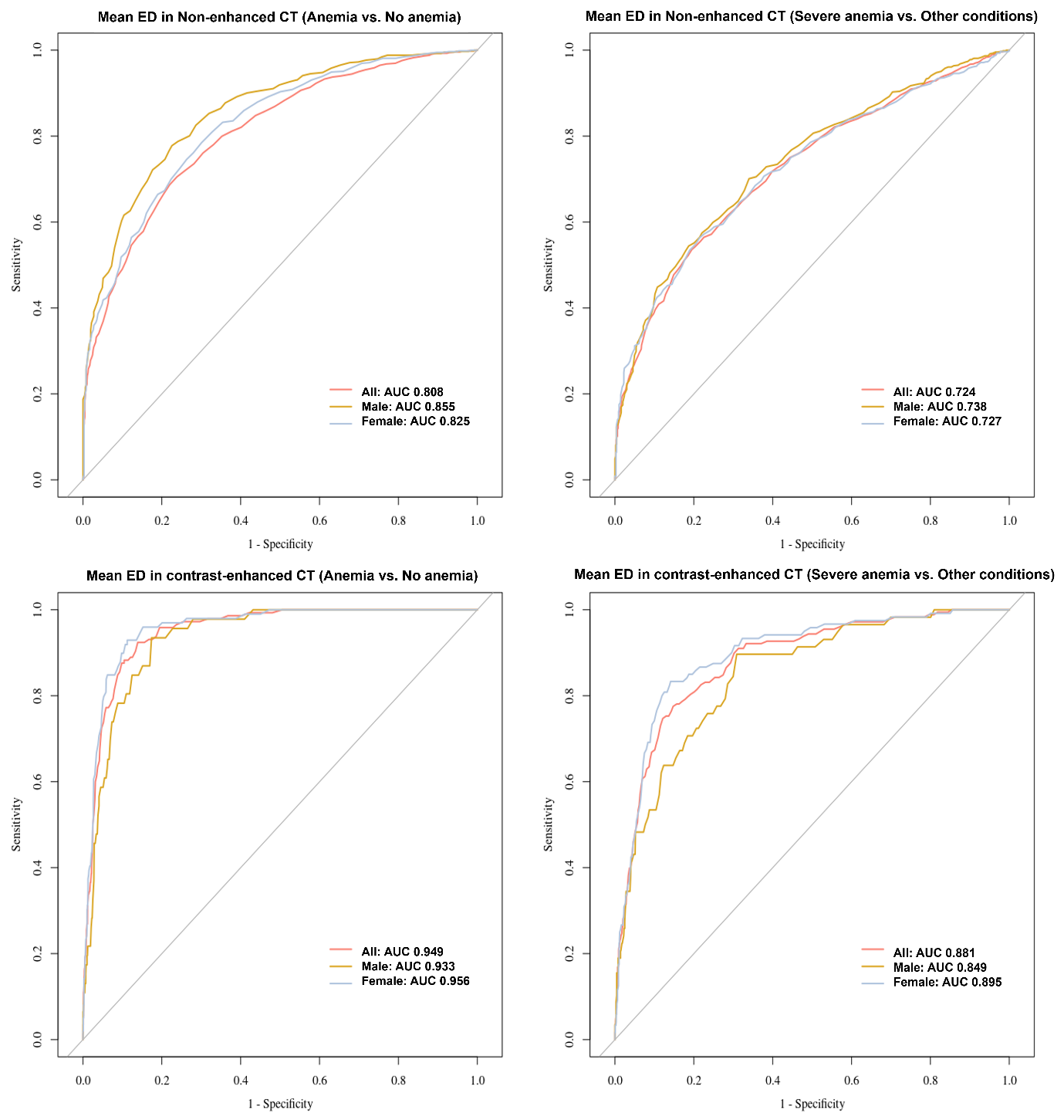

Supplement: S2 Fig — (DOCX) [file pone.0352504.s009.docx]
