# Supplementary material for: Evaluation of anemia in non-enhanced and contrast-enhanced dual-energy CT using electron density imaging
Source: PLoS One. 2026 Jul 2;21(7):e0352504. doi: 10.1371/journal.pone.0352504 (PMC13327118; doi:10.1371/journal.pone.0352504)
Supplement: Attachment — Submitted filename: authors reponses.docx [file pone.0352504.s012.docx]

**<Authors’ Responses>**

Additional Editor Comments:

Thank you for your message and for your patience regarding the review of your manuscript entitled “Evaluation of anemia in non-enhanced and contrast-enhanced dual-energy CT using electron density imaging” (Manuscript ID: PONE-D-25-42920).

We apologize for the delay in reaching a decision. During the peer review process, several invited reviewers declined to evaluate the manuscript, and in a number of instances these declinations were received close to the end of the review invitation period. As a result, additional rounds of reviewer invitations were required, which led to an unavoidable delay in completing the review process.

The peer review has now been completed. After careful evaluation of the reviewers’ reports, I agree with the recommendation that the manuscript requires major revision before it can be considered further for publication in PLOS ONE. In addition to the points raised by the reviewers, I have also identified a small number of minor editorial issues that will need to be addressed in the revision.

E1) Please justify the age 19 and above for selection.

R1) We thank the Academic Editor for these comments. The inclusion criterion of age ≥19 years was used to restrict the study population to adults. This approach ensured consistency with adult reference ranges for hemoglobin and hematocrit and avoided the known physiological variability of hematologic parameters in pediatric populations. We have now added this justification explicitly to the *Study Design* subsection of the Methods.

**[Revision in Materials and Methods]**

Study design

This retrospective study was approved by the institutional ethics review board (approval number: 2024AS0179), which granted a waiver of informed consent. No minors participated in the study. We identified 23,023 consecutive patients in our institution’s database who underwent either non-enhanced or contrast-enhanced chest CT using DECT between May 2019 and November 2023. The inclusion criteria were: 1) adults aged ≥19 years, because pediatric patients were excluded to avoid age-related differences in hematologic reference ranges and contrast enhancement characteristics; 2) individuals diagnosed with anemia or healthy individuals with normal Hb levels; and 3) those with Hb, Hct, and RBC count tests performed within 7 days before or after the CT scan.

E2) And all p-value should be written in small p letter.

R2) We carefully revised the entire manuscript to ensure that all p-values are consistently reported using a lowercase “p,” in accordance with journal style requirements.

**Comments to the Author**

Reviewer #1: This manuscript presents a large-scale, retrospective cohort study investigating the diagnostic utility of Electron Density (ED) maps derived from Dual-Energy Computed Tomography (DECT)—specifically employing dual-layer spectral detector CT (SDCT)—for the opportunistic assessment of anemia. The authors have analyzed a substantial dataset comprising 5,103 adult subjects, stratified into a non-enhanced CT (NECT) cohort of 2,558 patients and a contrast-enhanced CT (CECT) cohort of 2,545 patients. The primary objective was to validate ED as a robust surrogate biomarker for hemoglobin (Hb) and hematocrit (Hct) in the presence of iodinated contrast media, a scenario where conventional CT attenuation (Hounsfield Units, HU) traditionally fails due to the variable enhancement of the blood pool.

The context of this research is rooted in the evolving paradigm of opportunistic screening in radiology. Anemia is a prevalent comorbidity, particularly within hospital-based populations undergoing cross-sectional imaging for oncologic staging, trauma assessment, or acute abdominal pathology. It is well-established that anemia is an independent predictor of adverse outcomes, including increased perioperative mortality, prolonged hospitalization, and reduced quality of life. However, mild to moderate anemia often goes undiagnosed until it becomes symptomatic or severe.

While the manuscript addresses a high-value clinical question and leverages a robust sample size, there are significant reservations regarding the Technical Soundness of the authors' physical interpretation, the rigor of the Statistical Analysis, and compliance with Data Availability requirements. The assertion that ED is "independent" of contrast enhancement is physically imprecise and contradicted by the authors' own data, which shows a measurable shift in ED attributable to iodine. Furthermore, the submission fails to meet the PLOS ONE requirement for providing the "minimal data set" necessary for replication.

Therefore, I recommend a Major Revision.

Major Revisions:

QM1-1) Mandatory Data Availability Update: Your current Data Availability Statement is non-compliant with PLOS ONE policy. Summary tables (S1-S3) are insufficient for replication. You must provide the Minimal Data Set consisting of the individual data points (Age, Sex, Lab Values, CT Values) used to calculate the correlations and ROC curves. Please deposit this de-identified dataset in a public repository (e.g., Dryad, Figshare) or upload it as a supporting information file. The manuscript cannot be accepted without this data.

RM1-1) We fully agree with this important point and thank the reviewer for highlighting it. Accordingly, we provided a Data Set of the study data as supplementary material (S1 File).

- In accordance with the reviewer’s recommendation, we initially intended to provide the complete dataset, including the age variable, to ensure transparency and facilitate reproducibility of the analysis. However, during the editorial technical check, the PLOS ONE Editorial Office identified that the Supporting Information file contained potentially identifying information. Specifically, age was considered personally identifiable information under the journal’s data-sharing policy. Following the guidance from the Editorial Office, we have therefore removed the age variable and any potentially identifying information from the dataset. The revised Supporting Information file now contains only fully anonymized data that comply with the journal’s policies on participant privacy and data sharing. The anonymized dataset has been re-uploaded as the revised Supplementary Material. We hope that this revision addresses the concerns regarding data availability while ensuring compliance with the journal’s ethical and privacy requirements. We greatly appreciate the reviewer’s understanding.

QM1-2) Correction of Physical Interpretation: In the Discussion, you claim that "ED reflects tissue characteristics independently of contrast enhancement." This is contradicted by your own results (Table 2), which show that contrast administration increases the mean ED of non-anemic patients by approximately 1.6 units (from ~105.0 to ~106.6). This shift is larger than the difference between non-anemic and severely anemic patients (~1.2 units).

You must revise the text to acknowledge that iodine DOES contribute to electron density.

Explain that the utility of ED lies in the fact that the variance of this iodine contribution is low enough to preserve the anemia signal, whereas in HU, the iodine variance obliterates the signal.

RM1-2) We thank the reviewer for this critical and well-founded observation and fully agree. We revised the Discussion to correct the imprecise wording and now explicitly acknowledge that iodinated contrast media contribute additively to measured electron density. As the reviewer noted, our data show a measurable upward shift in mean ED after contrast administration. The revised manuscript clarifies that the key advantage of ED over HU in contrast-enhanced CT is not complete independence from iodine, but the lower variance of the iodine-related contribution to ED relative to HU. This lower variance preserves the anemia-related signal, whereas variability in iodine concentration in HU measurements obscures the relationship between attenuation and hemoglobin. This revision aligns the physical interpretation with our empirical findings (Table 2) and strengthens the study’s conceptual framework.

**[Revision in Discussion]**

Additionally, ED has been reported to be comparable to attenuation in single-energy CT (19, 20). Recent studies have suggested that ED values show no significant correlation with iodine concentration, implying that ED may reflect intrinsic tissue characteristics relatively independently of contrast enhancement (21). Our findings refine this interpretation. Although ED primarily reflects intrinsic tissue composition, our data indicate that iodinated contrast agents contribute additively to measured ED values. This effect is reflected in the systematic upward shift in mean ED in CECT compared with NECT. ED should therefore not be interpreted as completely independent of contrast enhancement. Rather, the diagnostic advantage of ED in CECT appears to arise from the relatively low variance of the iodine-related contribution. Although iodine increases absolute ED values, its variability appears sufficiently constrained to preserve the underlying anemia-related signal. This interpretation is supported by subanalysis across cardiac anatomical sites. Although correlations were lower in the pulmonary trunk and right ventricle than in the aorta and left ventricle, ED values maintained moderate correlations with hematologic parameters across all anatomical sites in CECT. In contrast, HU measurements in CECT are strongly influenced by variability in iodine concentration within the blood pool. This variability overwhelms attenuation differences attributable to Hb concentration and eliminates meaningful correlation with anemia severity. This distinction likely explains why ED, but not HU, retained diagnostic utility in CECT.

QM1-3) Hemodynamic Confounders: Please discuss the limitation of using a fixed 30-second scan delay. In patients with severe anemia, hyperdynamic circulation (high cardiac output) is common. This can lead to faster bolus transit, potentially reducing the iodine concentration in the aorta at 30 seconds compared to non-anemic patients. This hemodynamic variable is a confounder that might artificially lower ED in anemic patients (amplifying your signal) or raise it in heart failure patients (masking the signal).

RM1-3) We are sincerely grateful to the reviewer for this thoughtful and physiologically insightful comment. We agree that the use of a fixed 30-second scan delay introduces a potential hemodynamic confounder, particularly in conditions associated with altered cardiac output. In response, we have revised the Discussion section to explicitly acknowledge this limitation and to clarify the potential directionality of its effects. We further provide several considerations that reduce the likelihood that this confounder solely accounts for our findings: (1) independent validation of the ED–laboratory associations in the non-enhanced CT cohort, where iodine is absent; (2) the large and physiologically heterogeneous contrast-enhanced CT cohort (n = 2,545), which would be expected to introduce non-directional variability rather than systematic bias; and (3) the differential behavior of attenuation values, as HU measurements demonstrated near absence of correlation with laboratory parameters under identical acquisition and contrast conditions. We believe that these revisions improve the methodological transparency of the study and appropriately contextualize the potential influence of hemodynamic variability on our results.

**[Revision in Discussion]**

In the present study, a fixed 30-second scan delay was used for all contrast-enhanced CT examinations. Cardiac output is a major determinant of contrast bolus kinetics. High-output states—commonly observed in severe anemia as a compensatory mechanism—can accelerate contrast arrival and washout, whereas low-output states, such as heart failure, may delay peak aortic enhancement (25, 26). Consequently, at a uniform 30-second acquisition time, intravascular iodine concentration—and therefore its contribution to measured blood pool ED—may vary systematically according to individual hemodynamic status. In patients with severe anemia and compensatory hyperdynamic circulation, more rapid bolus transit may reduce aortic iodine concentration at the time of imaging (27), thereby directionally reinforcing ED reductions attributable to decreased RBC mass and potentially strengthening the observed associations. In contrast, in patients with reduced cardiac output, delayed peak enhancement may partially offset the expected ED decrease despite the presence of clinically significant anemia. Because cardiac output and related hemodynamic parameters were not available in this retrospective cohort, adjustment for this potential confounder was not feasible.

Nevertheless, several considerations mitigate the potential influence of this hemodynamic confounding. First, the moderate correlations observed between ED and laboratory parameters in the NECT cohort (r = 0.657–0.770). This finding provides independent evidence that ED intrinsically reflects RBC composition and supports the interpretation that the associations observed in the CECT cohort are not merely artifactual. Second, the large size of the CECT cohort (n = 2,545) includes patients with a wide range of physiological and cardiovascular conditions, encompassing both normal and impaired cardiac output states. In a population of this magnitude, inter-individual hemodynamic variability is more likely to contribute to stochastic variability rather than directional bias, thereby tending to dilute, rather than spuriously enhance, the strength of observed correlations. Third, the advantage of ED over conventional HU measurements in the contrast-enhanced setting is highlighted by the near absence of correlation between HU and laboratory parameters. This finding confirms that iodine predominantly determines HU values after contrast administration, rendering HU diagnostically unreliable for anemia detection. In contrast, ED maintained statistically significant and clinically meaningful correlations with Hb, Hct, and RBC count under identical acquisition conditions. The persistence of these associations despite shared exposure to contrast timing and potential hemodynamic variability suggests that ED retains partial functional independence from intravascular iodine concentration, consistent with its underlying physical basis in ED rather than HU. Future prospective studies incorporating bolus-tracking or individualized acquisition timing, along with documentation of hemodynamic variables, would help disentangle iodine-related variability from the intrinsic ED signal associated with RBC composition.

Minor Revisions:

Qm1-1) Bland-Altman Analysis: While Pearson's correlation is useful, it does not measure agreement. To validate the regression equations presented in Table 3 for clinical use, please consider adding a Bland-Altman plot to visualize the bias and limits of agreement between the "Predicted Hb" (derived from ED) and the "Actual Hb."

Rm1-1) We thank the reviewer for this helpful suggestion. We agree that Bland–Altman analysis provides information on agreement beyond correlation alone. Accordingly, we added Bland–Altman plots comparing ED-derived predicted hemoglobin, hematocrit, and red blood cell counts with laboratory-measured values. These results are now provided in a supplementary figure.

During the process of addressing the reviewer’s comments, we also identified and corrected an error in the formulas used in Table 3: the independent variable should have been ED rather than hemoglobin, hematocrit, or red blood cell counts. In addition, to reduce potential confusion, Figure 4 has been revised to display ED on the x-axis rather than the y-axis, and the figure has been redrawn accordingly.

**[Revision in Figure 4]**

New Figure 4 (A)

New Figure 4 (B)

**[Revision in Supplementary Figure 1]**

S1 Figure. Bland–Altman plots demonstrating agreement between laboratory-measured hemoglobin, hematocrit, and red blood cell counts and electron density (ED)–derived predicted values. (A) Contrast-enhanced CT. (B) Non-contrast CT.

(A)

(B)

Qm1-2) ROI Specificity: You averaged the ED values from the right and left heart chambers. Given the 30-second delay, the iodine concentration is likely heterogeneous between the Pulmonary Trunk and the Aorta. Please consider a sub-analysis to see if using only the Aortic or Left Ventricular ROIs yields a higher correlation with hemoglobin, as these chambers might be more stable at the 30-second time point.

Rm1-2) We sincerely thank the reviewer for this insightful comment regarding potential heterogeneity of iodine concentration across cardiac chambers at the 30-second post-contrast delay. Accordingly, we performed an additional subanalysis of correlations between hematologic parameters (hemoglobin, hematocrit, and RBC count) and both ED and HU across five anatomical locations: ascending aorta, descending aorta, pulmonary trunk, left ventricle, and right ventricle (S4 Table). Across all locations, ED demonstrated consistent moderate positive correlations with hematologic parameters. For hemoglobin, correlation coefficients ranged from r = 0.362 to r = 0.487 (all p < 0.001). The left ventricular ROI yielded the highest correlation (r = 0.487, p < 0.001), followed by the ascending and descending aorta (r = 0.459 and r = 0.457, respectively; both p < 0.001). In contrast, correlations were lower in the pulmonary trunk and right ventricle (r = 0.366 and r = 0.362, respectively), although they remained statistically significant. Conversely, HU showed weak or nonsignificant correlations across all locations, regardless of chamber selection. These findings suggest that ED-based measurements maintain stable correlations with hematologic parameters across vascular territories. Although left ventricular and aortic ROIs performed slightly better, the overall consistency across chambers supports the robustness of ED quantification and indicates that averaging across chambers does not materially bias the results.

**[Revision in Results]**

S4 Table summarizes the correlations between blood parameters and cardiac CT values at multiple anatomical sites in CECT. Across all sites, ED values showed consistent moderate positive correlations with hematologic parameters. For Hb, correlation coefficients ranged from r = 0.362 to r = 0.487 (all p < 0.001). The left ventricular ROI showed the strongest correlation (r = 0.487, p < 0.001), followed by the ascending and descending aorta (r = 0.459 and r = 0.457, respectively; both p < 0.001). Correlations were lower in the pulmonary trunk and right ventricle (r = 0.366 and r = 0.362, respectively) but remained statistically significant. By contrast, HU values showed weak or nonsignificant correlations across all anatomical sites, regardless of chamber selection.

**[Revision in Discussion]**

Additionally, ED has been reported to be comparable to attenuation in single-energy CT (19, 20). Recent studies have suggested that ED values show no significant correlation with iodine concentration, implying that ED may reflect intrinsic tissue characteristics relatively independently of contrast enhancement (21). Our findings refine this interpretation. Although ED primarily reflects intrinsic tissue composition, our data indicate that iodinated contrast agents contribute additively to measured ED values. This effect is reflected in the systematic upward shift in mean ED in CECT compared with NECT. ED should therefore not be interpreted as completely independent of contrast enhancement. Rather, the diagnostic advantage of ED in CECT appears to arise from the relatively low variance of the iodine-related contribution. Although iodine increases absolute ED values, its variability appears sufficiently constrained to preserve the underlying anemia-related signal. This interpretation is supported by subanalysis across cardiac anatomical sites. Although correlations were lower in the pulmonary trunk and right ventricle than in the aorta and left ventricle, ED values maintained moderate correlations with hematologic parameters across all anatomical sites in CECT. In contrast, HU measurements in CECT are strongly influenced by variability in iodine concentration within the blood pool. This variability overwhelms attenuation differences attributable to Hb concentration and eliminates meaningful correlation with anemia severity. This distinction likely explains why ED, but not HU, retained diagnostic utility in CECT.

Qm1-3) Abstract Clarification: Refine the sentence "mean HU significantly decreased only with NECT" to explicitly state that "mean HU in CECT showed no diagnostic correlation with anemia severity."

Rm1-3) We appreciate the reviewer’s comment. To improve clarity, we revised the Abstract to state explicitly that mean HU in CECT showed no statistically significant correlation with anemia severity, whereas significant correlations were observed in NECT.

**[Revision in Abstract]**

The mean ED showed strong positive correlations with Hb, Hct, and RBC count in both NECT and CECT (r = 0.657–0.770 for NECT and r = 0.447–0.583 for CECT, all p < 0.001), while mean HU significantly decreased only with NECT. In contrast, mean HU correlated with these parameters only in NECT and showed no significant diagnostic correlation in CECT.

Qm1-4) Generalizability: Please add a statement in the Discussion emphasizing that the specific ED cutoff values (e.g., 106.25 %EDW) are likely specific to the Philips IQon spectral reconstruction algorithm and may not be directly transferable to dual-source or rapid-kVp switching DECT platforms without cross-calibration.

Rm1-4) We thank the reviewer for this important comment regarding the platform-specific nature of ED cutoff values. As noted, our study was conducted using a single DECT scanner at one institution, and we had already acknowledged this as a limitation. In response to the reviewer’s suggestion, we revised the Discussion to clarify that the proposed ED cutoff values (e.g., 106.25 %EDW) are likely specific to the Philips IQon spectral reconstruction algorithm used in this study. These values may not be directly transferable to other DECT platforms, such as dual-source or rapid kVp-switching systems, without appropriate cross-calibration. This addition emphasizes the need for multicenter validation and cross-platform standardization before broader clinical implementation.

**[Revision in Discussion]**

Our study had several limitations. First, our DECT scans were conducted using a single specific CT scanner from one institution, which may limit the applicability of our cutoff values to scans performed with different machines from other vendors. Future research should incorporate multicenter data from various DECT machines produced by different manufacturers. First, our DECT scans were performed on a single-vendor platform at a single institution, which may limit the generalizability of our findings and the applicability of our cutoff values to other systems. In particular, the ED cutoff values identified in this study (e.g., 106.25% EDW for anemia detection) were derived using the Philips IQon dual-layer spectral detector CT and its proprietary spectral reconstruction algorithm. Because ED quantification and calibration methods vary across DECT technologies, including dual-source and rapid kVp-switching systems, these cutoff values may be platform-dependent and not directly transferable without cross-calibration and external validation. Future multicenter studies incorporating diverse DECT platforms are warranted to validate these findings and to establish vendor-neutral ED thresholds or standardized cross-platform calibration strategies. Second, our institutional protocol resulted in different radiation doses between NECT and CECT. In addition, information on clinical factors such as BMI, which could affect image quality, was unavailable. The extent to which ED values are influenced by radiation dose or image noise remains unclear, warranting further investigation.
