# Supplementary material for: Evaluation of anemia in non-enhanced and contrast-enhanced dual-energy CT using electron density imaging
Source: PLoS One. 2026 Jul 2;21(7):e0352504. doi: 10.1371/journal.pone.0352504 (PMC13327118; doi:10.1371/journal.pone.0352504)
Supplement: Attachment — Submitted filename: authors response_final.docx [file pone.0352504.s013.docx]

**Authors’ response**

We thank both reviewers for their thorough and constructive critique of our revised manuscript. We have addressed all comments in detail below. All corresponding revisions have been clearly indicated in the manuscript using the relevant notation numbers (e.g., R1-1, R2-3, etc.) to facilitate the reviewer’s reference.

Reviewer #1: The authors have addressed Reviewer 1's major concerns substantively. The revised discussion now correctly frames ED as not independent of iodine but rather having low iodine-related variance — a much stronger and more accurate argument. The hemodynamic confounder discussion is thorough. The Bland–Altman plots and site-specific subanalysis were appropriate additions. The formula errors in Table 3 were corrected. Data have been provided as S1 File, though with caveats (see below).

Major Comments

Q1-1) Incomplete minimal dataset (S1 File).
Upon inspection of the supplementary data, the file contains Sex, Hb, and per-site HU/ED values — but I could not identify columns for Hct or RBC count, which are key dependent variables in the correlation and ROC analyses. If these are truly absent, the dataset remains insufficient for full replication of the reported analyses. The authors should confirm and, if necessary, add these variables. Additionally, the removal of age — while understandable on privacy grounds — should be noted as a limitation for reproducibility, since age-stratified analyses cannot be replicated.

R1-1) We thank the reviewer for this careful inspection. We confirm that the uploaded S1 File inadvertently omitted the Hct and RBC count columns. We have now added these variables to the dataset. The file now includes: Sex, Hb, Hct, RBC count, mean ED per site, and mean HU per site for both NECT and CECT cohorts. We have also added a sentence to the S1 File description acknowledging that the removal of continuous age data limits full replication of any age-stratified analyses: “Individual-level age data were removed from the supplementary dataset on privacy grounds and replaced with age-group categories; readers wishing to replicate age-stratified subanalyses should be aware of this constraint.”

Q1-2) Correlation method discrepancy:
The abstract and Table 3 header state "Pearson's correlation coefficient," yet the revised Methods section now states that Spearman's rank correlation was used when normality was not met. It is unclear which method was actually applied for each analysis. The authors should explicitly state in the Results which coefficient was used for each comparison, or provide normality test results.
R1-2) We agree with the reviewer’s comment and appreciate the opportunity to clarify this point. In the revised manuscript, we have corrected the description to ensure full consistency between the Abstract and Table 3. Spearman’s rank correlation was the method applied for all primary correlations in Table 3. The wording in the Abstract and the whole main text has been revised to accurately reflect the correlation method applied in each analysis. All corresponding revisions have been indicated in the manuscript using the notation ‘R1-2.’

Q1-3) Clinical utility remains unclear for CECT.
The AUCs for detecting any-grade anemia on CECT are modest (0.73 males, 0.73 females) with sensitivities of 56–70% and specificities of 66–79%. These values are borderline for a screening tool. The Discussion should more explicitly acknowledge that while ED on CECT is statistically significant, its discriminatory performance for mild anemia may not be clinically actionable in isolation. As written, the conclusions somewhat overstate the CECT utility.
R1-3) We thank the reviewer for this insightful comment and fully agree that the discriminatory performance of ED on CECT for detecting any-grade anemia is modest and may not be clinically actionable in isolation, particularly for mild anemia. In response, we have revised the Discussion (including conclusion) to more explicitly address this limitation. Specifically, we now state that the AUCs for detecting any-grade anemia on CECT (0.727 in males; 0.738 in females) represent modest discriminatory performance, especially given that mild anemia constitutes the majority of detected cases, and that sensitivities of 56–70% and specificities of 66–79% at these operating points are unlikely to justify use as a stand-alone screening tool. We further clarify that the primary utility of ED in CECT is best conceptualized as opportunistic detection in patients already undergoing CECT for other indications, with particular relevance for severe anemia, for which AUCs exceeded 0.850–0.895 and sensitivities exceeded 77%. Finally, we emphasize that detection of mild anemia using ED on CECT should be interpreted as supplementary to, rather than a replacement for, laboratory testing.

[Revision in Discussion]

(…)

However, it is important to acknowledge that the AUCs for detecting any-grade anemia on CECT (0.727 in males; 0.738 in females) represent modest discriminatory performance, particularly given that mild anemia constitutes the majority of detected cases. At these operating points, sensitivities of 56–70% and specificities of 66–79% are unlikely to be clinically actionable as stand-alone screening evidence. The primary utility of ED in CECT is therefore best conceptualized as opportunistic detection — the recognition of unexpected or pre-existing anemia in patients already undergoing DECT for an unrelated clinical indication, such as oncology staging, pulmonary embolism workup, or trauma evaluation. This is particularly relevant for severe anemia, for which AUCs exceeded 0.850–0.895 and sensitivities exceeded 77%, representing a clinically meaningful level of discriminatory performance. In this opportunistic context, ED measurement requires no additional scan time, radiation exposure, or contrast administration, as the spectral data are inherently available from the standard acquisition. Automated flagging of reduced blood pool ED values within structured CT report templates or artificial intelligence-based post-processing workflows could prompt clinicians to order confirmatory laboratory testing at an earlier stage than they otherwise might, particularly in oncology patients at elevated risk for treatment-related anemia. Mild anemia detection using ED on CECT should be interpreted as supplementary to, not a replacement for, laboratory testing, and laboratory confirmation remains essential before any clinical decision-making. The feasibility and clinical impact of integrating ED-based anemia flagging into routine imaging workflows warrant prospective evaluation in future studies.

(…)

In conclusion, ED derived from DECT showed strong diagnostic utility for anemia detection on NECT and retained meaningful correlations with hematologic parameters on CECT, though its discriminatory performance for any-grade anemia on CECT was modest and should be interpreted as opportunistic detection rather than a stand-alone diagnostic tool. HU on NECT similarly reflected anemia severity, whereas HU on CECT showed no meaningful correlation with hematologic parameters.

Minor Comments

Q1-4) The manuscript still contains a duplicated sentence in the Limitations paragraph: "First, our DECT scans were conducted using a single specific CT scanner…" followed immediately by "First, our DECT scans were performed on a single-vendor platform…" — the old text was not fully removed (visible in the tracked-changes version, page 66, lines 402–414).

R1-4) We have reviewed the Limitations section and confirm that the duplicate sentence has been removed in the current revised manuscript.

Q1-5) Hemoglobin units are reported as "mg/dl" throughout, but the standard unit for hemoglobin is g/dL. This should be corrected.

R1-5) The reviewer is correct. This is an error that was carried throughout the manuscript. We have corrected all instances of “mg/dl” to “g/dL” throughout the manuscript, including Tables 1 and the anemia definition in the Methods. We apologize for this error. All corresponding revisions have been indicated in the manuscript using the notation ‘R1-5.’

Q1-6) Figure 3 legend states "(A) contrast-enhanced CT and (B) non-enhanced CT," but the uploaded images appear to show the reverse order based on the ED scale ranges (NECT ~103–106 in Fig 3B; CECT ~105–108 in Fig 3A). The authors should verify figure-label concordance.

R1-6) We thank the reviewer for this careful observation. We have re-examined the figure files and confirmed that the ED scale ranges are consistent with the correct assignment: Fig 3A shows CECT data (Mean ED ~104–108; Mean HU ~100–500) and Fig 3B shows NECT data (Mean ED ~102–106; Mean HU ~20–60). The figure panels are therefore correctly labeled as ‘(A) contrast-enhanced CT and (B) non-enhanced CT’ in the legend. No change to the figures or legend is required.

Q1-7) The S4 Table (site-specific CECT correlations) is referenced in the text but was not visible in the supporting materials I received for review. Please ensure it is included.

R1-7) We apologize for the oversight. Upon re-examination, we confirmed that S4 Table was included in the supplementary material file of the original submission; however, its legend had been inadvertently omitted from the Supporting Information section of the main manuscript. We have now added the corresponding legend for S4 Table to the Supporting Information section of the revised manuscript. Additionally, as noted in our response to Q1-2, Spearman’s rank correlation was applied throughout the analysis; accordingly, the correlation coefficient notation in S4 Table has been corrected from r to *r_s_*.

S4 Table. Spearman’s rank correlations between hematologic parameters and cardiac CT values by anatomical location in CECT.

|  | **Ascending aorta** | | **Pulmonary trunk** | | **Descending aorta** | | **Right ventricle** | | **Left ventricle** | |
| --- | --- | --- | --- | --- | --- | --- | --- | --- | --- | --- |
|  | **ED** | **HU** | **ED** | **HU** | **ED** | **HU** | **ED** | **HU** | **ED** | **HU** |
| ***Hb*** |  |  |  |  |  |  |  |  |  |  |
| *r_s_* | 0.459 | −0.029 | 0.366 | −0.077 | 0.457 | −0.005 | 0.362 | −0.059 | 0.487 | <0.001 |
| Significant level | <0.001 | 0.138 | <0.001 | <0.001 | <0.001 | 0.792 | <0.001 | 0.003 | <0.001 | 0.982 |
| ***Hct*** |  |  |  |  |  |  |  |  |  |  |
| *r_s_* | 0.439 | −0.024 | 0.351 | −0.074 | 0.439 | 0.001 | 0.339 | −0.063 | 0.466 | 0.006 |
| Significant level | <0.001 | 0.222 | <0.001 | <0.001 | <0.001 | 0.952 | <0.001 | 0.002 | <0.001 | 0.764 |
| ***RBC count*** |  |  |  |  |  |  |  |  |  |  |
| *r_s_* | 0.400 | −0.058 | 0.307 | −0.103 | 0.403 | −0.027 | 0.297 | −0.090 | 0.420 | −0.026 |
| Significant level | <0.001 | 0.003 | <0.001 | <0.001 | <0.001 | 0.177 | <0.001 | <0.001 | <0.001 | 0.183 |
| Note— CECT, contrast-enhanced CT; ED, electron density image; HU, CT attenuation; Hb, hemoglobin; Hct, hematocrit; RBC, red blood cell | | | | | | | | | | |

Reviewer #2:

Q2-1) Study Novelty and Clinical Impact
The concept of using CT-derived parameters for anemia detection is not entirely novel, as prior studies have evaluated HU and DECT-derived metrics.
The manuscript needs to better highlight what is new:
Is ED superior to VNC?
Is it clinically implementable in routine workflow?
Currently, the clinical impact is not strongly justified.
Suggestion: Clearly state how ED changes clinical decision-making beyond existing methods.

R2-1) We sincerely thank the reviewer for this thoughtful and constructive comment, which has helped us substantially strengthen the manuscript. We fully agree that the clinical impact of ED-based anemia detection requires clearer justification. We would like to respectfully clarify that the present study was not designed to directly compare ED with VNC reconstruction, and we therefore regret that we are unable to make direct claims regarding the superiority of ED over VNC at this time. We agree that a dedicated head-to-head comparison between ED and VNC for anemia detection in the CECT setting would be a highly valuable direction for future research, and we have noted this in the revised manuscript. We would, however, like to highlight an important conceptual distinction: VNC reconstruction mathematically removes the iodine signal entirely, whereas ED retains intrinsic tissue composition information and demonstrates relative robustness to iodine variability—preserving the anemia-related signal arising from RBC composition even under contrast-enhanced conditions. To our knowledge, no prior *in vivo* study has evaluated ED specifically for anemia detection in the CECT setting, and we believe this represents a meaningful contribution to the field.

Regarding the reviewer’s excellent suggestion to more clearly state how ED changes clinical decision-making beyond existing methods, we would like to draw the reviewer’s attention to the following passage already present in the Discussion: ‘Several studies have shown that VNC images from DECT or photon-counting CT can be used to detect and quantify anemia using CECT scans (12-14, 22). These findings, including ours, are clinically significant for oncology patients, who are particularly susceptible to anemia associated with treatments like chemotherapy or conditions such as cancer-associated bleeding. Given that cancer patients frequently undergo CECT scans for diagnostic or evaluative purposes, the ability to predict serum Hb, Hct, and RBC counts from routine CECT scans and perform early anemia evaluation could greatly aid in the timely detection and management of these complications. Consequently, CT imaging could become an even more valuable diagnostic tool in oncology practice.’

Furthermore, to more explicitly reflect both the clinical utility and limitations of ED-based anemia detection, we have revised the Conclusions as follows: ‘ED derived from DECT showed strong diagnostic utility for anemia detection on NECT and retained meaningful correlations with hematologic parameters on CECT, though its discriminatory performance for any-grade anemia on CECT was modest and should be interpreted as opportunistic detection rather than a stand-alone diagnostic tool. HU on NECT similarly reflected anemia severity, whereas HU on CECT showed no meaningful correlation with hematologic parameters.

We hope these clarifications and revisions adequately address the reviewer’s concerns and that the clinical relevance of our findings is now more clearly communicated. We remain grateful for the reviewer’s valuable guidance in improving the manuscript.

Q2-2) Study Design Limitations (Retrospective Nature)
The study is retrospective with no control over acquisition variability.
The 7-day window between CT and lab values may introduce temporal bias.
Concern: Hemoglobin levels can change rapidly, especially in hospitalized patients.
Suggestion:
Justify the 7-day window
Consider sensitivity analysis (e.g., ≤24-48 hours subgroup)
R2-2) We sincerely thank the reviewer for this constructive suggestion. We fully acknowledge that the retrospective nature of this study and the 7-day window between CT acquisition and laboratory testing represent potential limitations, particularly in hospitalized patients whose hemoglobin levels may fluctuate rapidly. Regarding the justification for the 7-day window: this interval was adopted to maximize cohort size while including patients whose clinical condition was sufficiently stable to allow meaningful correlation between hematologic parameters and CT-derived measurements. A narrower window would have substantially reduced the available sample size. In response to the reviewer’s suggestion, we performed a sensitivity analysis stratifying the cohort by the interval between CT and laboratory testing into three subgroups: less than 24 hours (0-24 hours), less than 48 hours (0-48 hours), and greater than 48 hours only. The results are summarized in the revised manuscript as Supplementary Table S5.

The Spearman’s rank correlation coefficients between mean ED and hematologic parameters were highly consistent across all temporal subgroups. For CECT, the correlations between mean ED and Hb were *r_s_* = 0.512 (0-24 hours) and *r_s_* = 0.508 (0-48 hours), compared with *r_s_* = 0.556 in the full cohort. Similar patterns were observed for Hct and RBC count. For NECT, correlations between mean ED and Hb were *r_s_* = 0.724 (0-24 hours) and *r_s_* = 0.724 (0-48 hours), compared with *r_s_* = 0.756 in the full cohort. Notably, the correlations in the shorter-interval subgroups were essentially equivalent to or slightly weaker than those in the full cohort, confirming that the 7-day inclusion window did not introduce meaningful temporal bias. By contrast, mean HU in CECT showed consistently negligible or weakly negative correlations with hematologic parameters across all subgroups, further supporting the robustness of our primary findings. We have added acknowledgment of this limitation and the sensitivity analysis results to the revised manuscript.

**[Revision in Results]**

In the sensitivity analysis stratified by CT-to-laboratory interval, Spearman’s rank correlation coefficients between mean ED and hematologic parameters remained consistent across all temporal subgroups in both CECT and NECT cohorts (S5 Table). For CECT, correlations between mean ED and Hb were *r_s_* = 0.512 and *r_s_* = 0.508 for the <24-hour and <48-hour subgroups, respectively, compared with *r_s_* = 0.556 in the full cohort. Similar trends were observed for Hct and RBC count. For NECT, correlations between mean ED and Hb were *r_s_* = 0.724 and *r_s_* = 0.724 for the <24-hour and <48-hour subgroups, respectively, compared with *r_s_* = 0.756 in the full cohort, with comparable consistency observed for Hct and RBC count. In contrast, mean HU in CECT showed consistently negligible or weakly negative correlations with all hematologic parameters across all temporal subgroups, consistent with the primary analysis.

**S5 Table**. Sensitivity analysis: Spearman’s rank correlation coefficients (*r_s_*) between CT measurements and hematologic parameters stratified by CT-to-laboratory interval.

| **NECT** | | |
| --- | --- | --- |
|  | **<24 hours (0-24 hours)** | **<48 hours (0-48 hours)** |
| **n** | **1,692** | **1,896** |
| ***Hb*** | | |
| Mean ED (*r_s_*) | 0.724 | 0.724 |
| Mean HU (*r_s_*) | 0.761 | 0.753 |
| ***Hct*** | | |
| Mean ED (*r_s_*) | 0.697 | 0.695 |
| Mean HU (*r_s_*) | 0.754 | 0.745 |
| ***RBC count*** | | |
| Mean ED (*r_s_*) | 0.651 | 0.65 |
| Mean HU (*r_s_*) | 0.699 | 0.694 |
| **CECT** | | |
|  | **<24 hours (0-24 hours)** | **<48 hours (0-48 hours)** |
| **n** | **1,739** | **1,947** |
| ***Hb*** | | |
| Mean ED (*r_s_*) | 0.512 | 0.508 |
| Mean HU (*r_s_*) | −0.002 | −0.016 |
| ***Hct*** | | |
| Mean ED (*r_s_*) | 0.489 | 0.484 |
| Mean HU (*r_s_*) | 0 | −0.013 |
| ***RBC count*** | | |
| Mean ED (*r_s_*) | 0.443 | 0.438 |
| Mean HU (*r_s_*) | −0.043 | −0.053* |

All correlations were statistically significant (*p* < 0.001) unless otherwise noted. **p* < 0.05 (not significant at *p* < 0.001 threshold). ED, electron density; HU, CT attenuation (Hounsfield unit); Hb, hemoglobin; Hct, hematocrit; RBC, red blood cell; CECT, contrast-enhanced CT; NECT, non-enhanced CT.

[Revision in Discussion (limitation)]

(…) Third, the 7-day window between CT acquisition and laboratory testing may introduce temporal bias, particularly in hospitalized patients in whom Hb levels can change rapidly. However, a pre-specified sensitivity analysis stratified by CT-to-laboratory interval demonstrated that correlation coefficients between mean ED and hematologic parameters were consistent across all subgroups for both CECT and NECT, supporting the robustness of the primary findings to this inclusion criterion. (…)

Q2-3) Confounding Variables Not Addressed
Important confounders are missing:
Hydration status
Cardiac output
Renal function
Contrast timing variability
BMI / body habitus
Although hemodynamic confounding is partially discussed, it is not quantified.
Impact: These factors may significantly influence ED and HU values.
R2-3) We sincerely thank the reviewer for this thoughtful comment. We fully agree that the confounding variables listed represent important limitations of this study, and we appreciate the opportunity to address them more explicitly. We acknowledge that hydration status, cardiac output, renal function, contrast timing variability, and BMI/body habitus may all influence blood pool ED and HU measurements to varying degrees. In the context of our retrospective single-center study, these variables were not consistently available in the clinical database, and their independent quantitative contributions to measured ED values could therefore not be determined. Regarding hemodynamic confounding specifically, we have already discussed in detail in the Discussion the potential influence of cardiac output on contrast bolus kinetics and its implications for blood pool ED measurement under the fixed 30-second acquisition protocol. While we agree that quantification of this effect would strengthen the interpretation of our findings, such analysis was not feasible given the retrospective design and the unavailability of cardiac output data in this cohort. Regarding BMI and body habitus, we acknowledge that these factors may affect image noise and potentially influence ED measurements, particularly in larger patients. Unfortunately, BMI data were not consistently recorded in association with the CT acquisitions in our dataset, which we have noted as a limitation. In response to this comment, we have expanded the Limitations section of the revised manuscript to enumerate these confounders explicitly.

[Revision in Discussion (limitation)]

(…) Fourth, additional unmeasured confounders that may influence blood pool ED and HU measurements include hydration status, renal function, BMI and body habitus, and contrast timing variability arising from differences in cardiac output and venous access. Because these variables were not consistently available in our retrospective dataset, their independent contributions to measured ED values could not be quantified. Future prospective studies should incorporate these variables into a multivariate framework to better isolate the contribution of hematologic parameters to blood pool ED, and to determine whether adjustment for hemodynamic and physiological confounders meaningfully alters the observed associations.

Q2-4) Statistical Analysis Concerns
Reliance on correlation (r values) is insufficient.
Correlation ≠ clinical agreement.
Issues:
No clear multivariate analysis
No adjustment for confounders
Regression models are not validated externally
Suggestion:
Add multivariate regression
Provide model performance (R², RMSE)
Include internal validation (bootstrap or split-sample)

R2-4) We sincerely thank the reviewer for this important methodological comment. We agree that correlation coefficients alone provide an incomplete picture of the relationship between CT-derived measurements and hematologic parameters, and we have taken several steps to address this concern.

First, regarding the suggestion to add multivariate analysis with adjustment for confounders: we performed partial correlation analysis adjusting for age and sex—the two key demographic confounders available in our dataset—using Spearman’s rank partial correlation. The results are presented in S6 Table. After adjustment for age and sex, partial correlation coefficients between mean ED and Hb remained substantial for both NECT (*r_s_* = 0.694, full cohort) and CECT (*r_s_* = 0.509, full cohort), and were highly consistent across all temporal subgroups, confirming that the observed associations are not explained by demographic confounding.

Second, regarding model performance metrics: the reviewer suggests providing *R*^2^ and RMSE as indicators of model performance. We would like to respectfully clarify that the primary analytical framework of this study is correlation analysis, which quantifies the strength of association between two variables rather than the predictive accuracy of a regression model. In this context, RMSE is a metric specific to regression-based prediction models and cannot be meaningfully derived from correlation analysis alone. We therefore do not consider it appropriate to report RMSE in the absence of a formal regression prediction framework. However, in response to the reviewer’s suggestion, we have calculated R^2^ as the square of the partial correlation coefficient (shown in S6 Table), which represents the proportion of variance in hematologic parameters explained by mean ED after adjusting for age and sex. For NECT, mean ED explained 48.2% of the variance in Hb (R^2^ = 0.482), 44.1% in Hct (R^2^ = 0.441), and 37.6% in RBC count (R^2^ = 0.376). For CECT, mean ED explained 25.9% of the variance in Hb (R^2^ = 0.259), 23.3% in Hct (R^2^ = 0.233), and 18.9% in RBC count (R^2^ = 0.189). These values reflect the inherent variability introduced by contrast enhancement and unmeasured physiological confounders, and are consistent with the known limitations of opportunistic anemia detection in the CECT setting.

Furthermore, to address the reviewer’s suggestion regarding internal validation, we performed a nonparametric bootstrap analysis (1,000 resamples with replacement) to assess the stability and reproducibility of the partial correlation coefficients after adjustment for age and sex. The bootstrap analysis yielded negligible bias for all estimates—for example, the bias for the partial correlation between mean ED and Hb was −0.0004 in CECT and −0.0001 in NECT, both of which are trivially small relative to the point estimates—confirming the absence of meaningful overfitting. The bootstrap-derived 95% confidence intervals were narrow and did not include zero for mean ED across all hematologic parameters in both CECT and NECT, further supporting the robustness of the observed associations. For CECT, the bootstrap 95% CIs for mean ED were *r_s_* = 0.509 (95% CI, 0.477-0.538) for Hb, *r_s_* = 0.482 (95% CI, 0.450-0.513) for Hct, and *r_s_* = 0.435 (95% CI, 0.400-0.467) for RBC count. For NECT, the corresponding values were *r_s_* = 0.694 (95% CI, 0.671-0.717) for Hb, *r_s_* = 0.664 (95% CI, 0.639-0.688) for Hct, and *r_s_* = 0.613 (95% CI, 0.587-0.641) for RBC count. In contrast, the bootstrap 95% CI for mean HU in CECT crossed or approached zero for RBC count (*r_s_* = 0.017, 95% CI, −0.025-0.054), consistent with the absence of a meaningful association between HU and hematologic parameters in the contrast-enhanced setting. These bootstrap results have been added to the S7 Table in the revised manuscript.

[Revision in Results and Supplementary Tables]

To adjust for potential demographic confounding, partial correlation analysis controlling for age and sex was performed. After adjustment, partial correlation coefficients between mean ED and Hb remained substantial in both NECT (rs = 0.694, R² = 0.482) and CECT (rs = 0.509, R² = 0.259), and were consistent across all CT-to-laboratory interval subgroups (S6 Table). These findings indicate that the associations between mean ED and hematologic parameters are not attributable to demographic confounding.

In addition, bootstrap internal validation (1,000 resamples) of the age- and sex-adjusted partial correlation coefficients demonstrated negligible bias and narrow 95% confidence intervals for all mean ED estimates in both CECT and NECT, confirming the stability of the observed associations (S7 Table). By contrast, the bootstrap 95% confidence interval (CI) for mean HU in CECT crossed zero for RBC count (95% CI −0.025–0.054), further confirming the absence of a clinically meaningful association between HU and hematologic parameters in the contrast-enhanced setting.

**S6 Table**. Partial correlation coefficients (*r_s_*) and coefficients of determination (R^2^) between mean ED/mean HU and hematologic parameters, adjusted for age and sex, stratified by CT-to-laboratory interval.

| **NECT** | | | | |
| --- | --- | --- | --- | --- |
| **Parameter** | **CT measurements** | **All** | **<24 hours (0-24 hours)** | **<48 hours (0-48 hours)** |
| ***Hb*** | **Mean ED** | *r_s_* = 0.694 | *r_s_* = 0.713 | *r_s_* = 0.709 |
|  |  | R^2^ = 0.482 | R^2^ = 0.508 | R^2^ = 0.502 |
|  | **Mean HU** | *r_s_* = 0.735 | *r_s_* = 0.758 | *r_s_* = 0.749 |
|  |  | R^2^ = 0.540 | R^2^ = 0.575 | R^2^ = 0.561 |
| ***Hct*** | **Mean ED** | *r_s_* = 0.664 | *r_s_* = 0.684 | *r_s_* = 0.678 |
|  |  | R^2^ = 0.441 | R^2^ = 0.468 | R^2^ = 0.460 |
|  | **Mean HU** | *r_s_* = 0.725 | *r_s_* = 0.749 | *r_s_* = 0.738 |
|  |  | R^2^ = 0.526 | R^2^ = 0.561 | R^2^ = 0.545 |
| ***RBC count*** | **Mean ED** | *r_s_* = 0.613 | *r_s_* = 0.635 | *r_s_* = 0.631 |
|  |  | R^2^ = 0.376 | R^2^ = 0.403 | R^2^ = 0.398 |
|  | **Mean HU** | *r_s_* = 0.668 | *r_s_* = 0.688 | *r_s_* = 0.679 |
|  |  | R^2^ = 0.446 | R^2^ = 0.473 | R^2^ = 0.461 |
| **CECT** | | | | |
| **Parameter** | **Metric** | **All** | **<24 hours (0-24 hours)** | **<48 hours (0-48 hours)** |
| ***Hb*** | **Mean ED** | *r_s_* = 0.509 | *r_s_* = 0.532 | *r_s_* = 0.526 |
|  |  | R^2^ = 0.259 | R^2^ = 0.283 | R^2^ = 0.277 |
|  | **Mean HU** | *r_s_* = 0.055 | *r_s_* = 0.080 | *r_s_* = 0.078 |
|  |  | R^2^ = 0.003 | R^2^ = 0.006 | R^2^ = 0.006 |
| ***Hct*** | **Mean ED** | *r_s_* = 0.482 | *r_s_* = 0.507 | *r_s_* = 0.500 |
|  |  | R^2^ = 0.233 | R^2^ = 0.257 | R^2^ = 0.250 |
|  | **Mean HU** | *r_s_* = 0.054 | *r_s_* = 0.079 | *r_s_* = 0.077 |
|  |  | R^2^ = 0.003 | R^2^ = 0.006 | R^2^ = 0.006 |
| ***RBC count*** | **Mean ED** | *r_s_* = 0.435 | *r_s_* = 0.461 | *r_s_* = 0.453 |
|  |  | R^2^ = 0.189 | R^2^ = 0.212 | R^2^ = 0.205 |
|  | **Mean HU** | *r_s_* = 0.017 | *r_s_* = 0.035 | *r_s_* = 0.035 |
|  |  | R^2^ = 0.000 | R^2^ = 0.001 | R^2^ = 0.001 |

All correlations were statistically significant (*p* < 0.001) unless otherwise noted. Partial correlations were adjusted for age and sex. R^2^ was calculated as the square of the partial Spearman’s rank correlation coefficient (*r*_s_^2^), representing the proportion of variance in hematologic parameters explained by the CT measurement after demographic adjustment. ED, electron density; HU, Hounsfield unit; Hb, hemoglobin; Hct, hematocrit; RBC, red blood cell; CECT, contrast-enhanced CT; NECT, non-enhanced CT.

**S7 Table**. Bootstrap internal validation (1,000 resamples) of partial correlation coefficients (*r_s_*) between mean ED/mean HU and hematologic parameters, adjusted for age and sex.

| **NECT** | | | | | | | |
| --- | --- | --- | --- | --- | --- | --- | --- |
| **Parameter** | **CT measurements** | **Estimate (*r_s_*)** | **Bias** | **SE** | **95% CI Lower** | **95% CI Upper** | **R^2^** |
| ***Hb*** | **Mean ED** | 0.694 | −0.0001 | 0.0117 | 0.671 | 0.717 | 0.482 |
|  | **Mean HU** | 0.735 | −0.0004 | 0.0111 | 0.713 | 0.756 | 0.540 |
| ***Hct*** | **Mean ED** | 0.664 | −0.0002 | 0.0125 | 0.639 | 0.688 | 0.441 |
|  | **Mean HU** | 0.725 | −0.0003 | 0.0113 | 0.702 | 0.748 | 0.526 |
| ***RBC count*** | **Mean ED** | 0.613 | 0.0005 | 0.0135 | 0.587 | 0.641 | 0.376 |
|  | **Mean HU** | 0.668 | 0.0003 | 0.0128 | 0.642 | 0.693 | 0.446 |
| **CECT** | | | | | | | |
| **Parameter** | **CT measurements** | **Estimate (*r_s_*)** | **Bias** | **SE** | **95% CI Lower** | **95% CI Upper** | **R^2^** |
| ***Hb*** | **Mean ED** | 0.509 | −0.0004 | 0.0157 | 0.477 | 0.538 | 0.259 |
|  | **Mean HU** | 0.055 | −0.0007 | 0.0202 | 0.015 | 0.093 | 0.003 |
| ***Hct*** | **Mean ED** | 0.482 | −0.0002 | 0.0163 | 0.450 | 0.513 | 0.233 |
|  | **Mean HU** | 0.054 | −0.0006 | 0.0205 | 0.011 | 0.092 | 0.003 |
| ***RBC count*** | **Mean ED** | 0.435 | −0.0002 | 0.017 | 0.400 | 0.467 | 0.189 |
|  | **Mean HU** | 0.017* | −0.0007 | 0.0204 | **−**0.025***** | 0.054 | 0 |

Bootstrap analysis performed using ordinary nonparametric bootstrap with 1,000 resamples. Partial correlations were adjusted for age and sex. SE, standard error; CI, confidence interval; *R^2^ = r_s_^2^, representing the proportion of variance explained after demographic adjustment.* Values with * indicate 95% CI crossing zero (i.e., non-significant association). ED, electron density; HU, Hounsfield unit; Hb, hemoglobin; Hct, hematocrit; RBC, red blood cell; CECT, contrast-enhanced CT; NECT, non-enhanced CT.

Q2-5) Lack of External Validation
Single-center study using a single scanner (Philips IQon)
ED values are vendor-dependent

Impact: Limits generalizability
Suggestion:
Explicitly emphasize this limitation
Avoid overgeneralizing cutoff values

R2-5) We sincerely thank the reviewer for this important comment. We fully agree that the single-center, single-vendor design of this study represents a significant limitation, and we appreciate the opportunity to address this more explicitly. ED quantification is inherently dependent on the spectral reconstruction algorithm employed by each DECT platform. The Philips IQon dual-layer spectral detector CT used in this study generates ED maps through a proprietary reconstruction pipeline that may differ substantially from those of other DECT technologies, including dual-source systems (Siemens) and rapid kVp-switching systems (GE). As a result, the absolute ED values and diagnostic cutoff values reported in this study—for example, 106.25 %EDW for any-grade anemia detection on CECT—are platform-specific and should not be directly applied to other scanner types without cross-calibration and independent validation.

In response to the reviewer’s suggestion, we have substantially expanded the Limitations section of the revised manuscript to emphasize this point more explicitly, as follows: ‘First, this study was conducted at a single institution using a single DECT platform (Philips IQon dual-layer spectral detector CT), which limits the generalizability of our findings. ED quantification methods and calibration standards vary across DECT technologies, and the diagnostic cutoff values identified in this study are therefore platform-dependent and cannot be directly transferred to other scanner types without cross-calibration and external validation. Readers are cautioned against overgeneralizing the reported cutoff values beyond the specific acquisition and reconstruction conditions of this study. Future multicenter studies incorporating diverse DECT platforms—including dual-source and rapid kVp-switching systems—are warranted to establish vendor-neutral ED thresholds and to confirm the generalizability of the observed associations across different imaging environments.’

Additionally, we have moderated the language in the Abstract to avoid overgeneralization, replacing ‘ED is a valuable tool’ with ‘ED-based anemia detection on CECT is best conceptualized as opportunistic detection, particularly for severe anemia, supplementary to laboratory testing. These findings require external validation across diverse DECT platforms before broader clinical implementation.’

We hope these revisions adequately address the reviewer’s concerns regarding the generalizability of our findings.

Q2-6) Interpretation of ED and Contrast Effects
The manuscript initially suggests ED is independent of contrast, but data show otherwise.
 Even after revision, interpretation remains overstated.
 Key issue:
ED is less affected, not independent.
 Suggestion:
Use cautious wording such as:
“ED demonstrates relative robustness to contrast variation rather than independent

R2-6) We sincerely thank the reviewer for this precise and valuable comment. Upon careful re-examination of the manuscript, we identified one instance where the phrasing may have given an impression of contrast independence. In the Discussion section, we had written: ‘Recent studies have suggested that ED values show no significant correlation with iodine concentration, implying that ED may reflect intrinsic tissue characteristics relatively independently of contrast enhancement (21).’ We would like to clarify that this sentence was intended to summarize the interpretation proposed in prior literature, and was immediately followed by ‘Our findings refine this interpretation,‘ after which we explicitly stated that ‘ED should therefore not be interpreted as completely independent of contrast enhancement.’ The overall intent of the paragraph was therefore to correct, rather than endorse, the notion of contrast independence.

Nevertheless, we fully appreciate that even in the context of citing prior literature, the use of the phrase ‘relatively independently of contrast enhancement’ may cause unintended misinterpretation among readers, and we are grateful to the reviewer for drawing our attention to this. To minimize any potential ambiguity and to ensure that the cautious interpretation is conveyed from the outset of the paragraph, we have revised the sentence as follows: ‘Recent studies have suggested that ED values show no significant correlation with iodine concentration, implying that ED may reflect intrinsic tissue characteristics with relative robustness to contrast enhancement (21).’ We believe this revision, in conjunction with the clarifying sentences that follow, now presents a consistent and appropriately nuanced interpretation of the relationship between ED and contrast enhancement throughout the manuscript.

Q2-7) Clinical Applicability is Unclear
How would ED-based anemia detection be used?
Screening?
Opportunistic detection?
Replacement for lab tests?

Concern: No workflow integration discussed

R2-7) We sincerely thank the reviewer for this clinically important and thoughtful comment. We fully appreciate that the lack of explicit discussion regarding clinical workflow integration represents an important gap in the original manuscript, and we are genuinely grateful to the reviewer for highlighting this point.

In response to the reviewer’s concern, we have carefully revised and expanded the relevant passage in the Discussion section to explicitly address how ED-based anemia detection would be used in clinical practice, its relationship to existing laboratory testing, and its potential integration into clinical workflows. The revised passage now reads as follows:

‘The primary utility of ED in CECT is therefore best conceptualized as opportunistic detection—the recognition of unexpected or pre-existing anemia in patients already undergoing DECT for an unrelated clinical indication, such as oncology staging, pulmonary embolism workup, or trauma evaluation. This is particularly relevant for severe anemia, for which AUCs exceeded 0.85-0.90, and sensitivities exceeded 77%, representing a clinically meaningful level of discriminatory performance. In this opportunistic context, ED measurement requires no additional scan time, radiation exposure, or contrast administration, as the spectral data are inherently available from the standard acquisition. Automated flagging of reduced blood pool ED values within structured CT report templates or artificial intelligence-based post-processing workflows could prompt clinicians to order confirmatory laboratory testing at an earlier stage than they otherwise might, particularly in oncology patients at elevated risk for treatment-related anemia. Mild anemia detection using ED on CECT should be interpreted as supplementary to, not a replacement for, laboratory testing, and laboratory confirmation remains essential before any clinical decision-making. The feasibility and clinical impact of integrating ED-based anemia flagging into routine imaging workflows warrant prospective evaluation in future studies.’

To directly address each of the reviewer’s specific questions: ED-based anemia detection is proposed exclusively as an opportunistic detection tool, not as a population-level screening program. It is not intended to replace serum hemoglobin measurement or other laboratory tests; rather, it serves as a flag within an existing imaging workflow to prompt timely confirmatory laboratory testing. We hope that these revisions now provide the clarity the reviewer sought, and we remain grateful for this valuable guidance in strengthening the clinical relevance of our manuscript.
