# Supplementary material for: Evaluation of anemia in non-enhanced and contrast-enhanced dual-energy CT using electron density imaging
Source: PLoS One. 2026 Jul 2;21(7):e0352504. doi: 10.1371/journal.pone.0352504 (PMC13327118; doi:10.1371/journal.pone.0352504)
Supplement: Peer review history

# Peer review: Decision Letter 0

**DOI:** 10.1371/journal.pone.0352504.r001

**Authors:** M Tanveer Hossain Parash

28 Jan 2026

PONE-D-25-42920

Evaluation of anemia in non-enhanced and contrast-enhanced dual-energy CT using electron density imaging

PLOS One

Dear Dr. Kim,

Thank you for submitting your manuscript to PLOS ONE. After careful consideration, we feel that it has merit but does not fully meet PLOS ONE’s publication criteria as it currently stands. Therefore, we invite you to submit a revised version of the manuscript that addresses the points raised during the review process.

Please submit your revised manuscript by Mar 14 2026 11:59PM. If you will need more time than this to complete your revisions, please reply to this message or contact the journal office at plosone@plos.org. When you're ready to submit your revision, log on to https://www.editorialmanager.com/pone/ and select the 'Submissions Needing Revision' folder to locate your manuscript file.

Please include the following items when submitting your revised manuscript:

- A letter that responds to each point raised by the academic editor and reviewer(s). You should upload this letter as a separate file labeled 'Response to Reviewers'.
- A marked-up copy of your manuscript that highlights changes made to the original version. You should upload this as a separate file labeled 'Revised Manuscript with Track Changes'.
- An unmarked version of your revised paper without tracked changes. You should upload this as a separate file labeled 'Manuscript'.

If you would like to make changes to your financial disclosure, please include your updated statement in your cover letter. Guidelines for resubmitting your figure files are available below the reviewer comments at the end of this letter.

If applicable, we recommend that you deposit your laboratory protocols in protocols.io to enhance the reproducibility of your results. Protocols.io assigns your protocol its own identifier (DOI) so that it can be cited independently in the future. For instructions see: https://journals.plos.org/plosone/s/submission-guidelines#loc-laboratory-protocols. Additionally, PLOS ONE offers an option for publishing peer-reviewed Lab Protocol articles, which describe protocols hosted on protocols.io. Read more information on sharing protocols at https://plos.org/protocols?utm_medium=editorial-email&utm_source=authorletters&utm_campaign=protocols.

We look forward to receiving your revised manuscript.

Kind regards,

M Tanveer Hossain Parash, MBBS, M Phil

Academic Editor

PLOS One

Journal Requirements:

When submitting your revision, we need you to address these additional requirements.

1.Please ensure that your manuscript meets PLOS ONE's style requirements, including those for file naming. The PLOS ONE style templates can be found at

https://journals.plos.org/plosone/s/file?id=wjVg/PLOSOne_formatting_sample_main_body.pdf and

https://journals.plos.org/plosone/s/file?id=ba62/PLOSOne_formatting_sample_title_authors_affiliations.pdf

2. We note that the grant information you provided in the ‘Funding Information’ and ‘Financial Disclosure’ sections do not match.

When you resubmit, please ensure that you provide the correct grant numbers for the awards you received for your study in the ‘Funding Information’ section.

3. If the reviewer comments include a recommendation to cite specific previously published works, please review and evaluate these publications to determine whether they are relevant and should be cited. There is no requirement to cite these works unless the editor has indicated otherwise.

Additional Editor Comments:

Thank you for your message and for your patience regarding the review of your manuscript entitled “Evaluation of anemia in non-enhanced and contrast-enhanced dual-energy CT using electron density imaging” (Manuscript ID: PONE-D-25-42920).

We apologize for the delay in reaching a decision. During the peer review process, several invited reviewers declined to evaluate the manuscript, and in a number of instances these declinations were received close to the end of the review invitation period. As a result, additional rounds of reviewer invitations were required, which led to an unavoidable delay in completing the review process.

The peer review has now been completed. After careful evaluation of the reviewers’ reports, I agree with the recommendation that the manuscript requires major revision before it can be considered further for publication in PLOS ONE. In addition to the points raised by the reviewers, I have also identified a small number of minor editorial issues that will need to be addressed in the revision.

Please justify the age 19 and above for selection.

And all p-value should be written in small p letter.

[Note: HTML markup is below. Please do not edit.]

Reviewer's Responses to Questions

**Comments to the Author**

1. Is the manuscript technically sound, and do the data support the conclusions?

The manuscript must describe a technically sound piece of scientific research with data that supports the conclusions. Experiments must have been conducted rigorously, with appropriate controls, replication, and sample sizes. The conclusions must be drawn appropriately based on the data presented.

Reviewer #1: Partly

**********

2. Has the statistical analysis been performed appropriately and rigorously?

Reviewer #1: Yes

**********

3. Have the authors made all data underlying the findings in their manuscript fully available?

The PLOS Data policy requires authors to make all data underlying the findings described in their manuscript fully available without restriction, with rare exception (please refer to the Data Availability Statement in the manuscript PDF file). The data should be provided as part of the manuscript or its supporting information, or deposited to a public repository. For example, in addition to summary statistics, the data points behind means, medians and variance measures should be available. If there are restrictions on publicly sharing data—e.g. participant privacy or use of data from a third party—those must be specified.

Reviewer #1: No

**********

4. Is the manuscript presented in an intelligible fashion and written in standard English?

PLOS ONE does not copyedit accepted manuscripts, so the language in submitted articles must be clear, correct, and unambiguous. Any typographical or grammatical errors should be corrected at revision, so please note any specific errors here.

Reviewer #1: Yes

**********

5. Review Comments to the Author

Please use the space provided to explain your answers to the questions above. You may also include additional comments for the author, including concerns about dual publication, research ethics, or publication ethics. (Please upload your review as an attachment if it exceeds 20,000 characters)

Reviewer #1: This manuscript presents a large-scale, retrospective cohort study investigating the diagnostic utility of Electron Density (ED) maps derived from Dual-Energy Computed Tomography (DECT)—specifically employing dual-layer spectral detector CT (SDCT)—for the opportunistic assessment of anemia. The authors have analyzed a substantial dataset comprising 5,103 adult subjects, stratified into a non-enhanced CT (NECT) cohort of 2,558 patients and a contrast-enhanced CT (CECT) cohort of 2,545 patients. The primary objective was to validate ED as a robust surrogate biomarker for hemoglobin (Hb) and hematocrit (Hct) in the presence of iodinated contrast media, a scenario where conventional CT attenuation (Hounsfield Units, HU) traditionally fails due to the variable enhancement of the blood pool.

The context of this research is rooted in the evolving paradigm of opportunistic screening in radiology. Anemia is a prevalent comorbidity, particularly within hospital-based populations undergoing cross-sectional imaging for oncologic staging, trauma assessment, or acute abdominal pathology. It is well-established that anemia is an independent predictor of adverse outcomes, including increased perioperative mortality, prolonged hospitalization, and reduced quality of life. However, mild to moderate anemia often goes undiagnosed until it becomes symptomatic or severe.

While the manuscript addresses a high-value clinical question and leverages a robust sample size, there are significant reservations regarding the Technical Soundness of the authors' physical interpretation, the rigor of the Statistical Analysis, and compliance with Data Availability requirements. The assertion that ED is "independent" of contrast enhancement is physically imprecise and contradicted by the authors' own data, which shows a measurable shift in ED attributable to iodine. Furthermore, the submission fails to meet the PLOS ONE requirement for providing the "minimal data set" necessary for replication.

Therefore, I recommend a Major Revision.

Major Revisions:

(1) Mandatory Data Availability Update: Your current Data Availability Statement is non-compliant with PLOS ONE policy. Summary tables (S1-S3) are insufficient for replication. You must provide the Minimal Data Set consisting of the individual data points (Age, Sex, Lab Values, CT Values) used to calculate the correlations and ROC curves. Please deposit this de-identified dataset in a public repository (e.g., Dryad, Figshare) or upload it as a supporting information file. The manuscript cannot be accepted without this data.

(2) Correction of Physical Interpretation: In the Discussion, you claim that "ED reflects tissue characteristics independently of contrast enhancement." This is contradicted by your own results (Table 2), which show that contrast administration increases the mean ED of non-anemic patients by approximately 1.6 units (from ~105.0 to ~106.6). This shift is larger than the difference between non-anemic and severely anemic patients (~1.2 units).

You must revise the text to acknowledge that iodine DOES contribute to electron density.

Explain that the utility of ED lies in the fact that the variance of this iodine contribution is low enough to preserve the anemia signal, whereas in HU, the iodine variance obliterates the signal.

(3) Hemodynamic Confounders: Please discuss the limitation of using a fixed 30-second scan delay. In patients with severe anemia, hyperdynamic circulation (high cardiac output) is common. This can lead to faster bolus transit, potentially reducing the iodine concentration in the aorta at 30 seconds compared to non-anemic patients. This hemodynamic variable is a confounder that might artificially lower ED in anemic patients (amplifying your signal) or raise it in heart failure patients (masking the signal).

Minor Revisions:

(1) Bland-Altman Analysis: While Pearson's correlation is useful, it does not measure agreement. To validate the regression equations presented in Table 3 for clinical use, please consider adding a Bland-Altman plot to visualize the bias and limits of agreement between the "Predicted Hb" (derived from ED) and the "Actual Hb."

(2) ROI Specificity: You averaged the ED values from the right and left heart chambers. Given the 30-second delay, the iodine concentration is likely heterogeneous between the Pulmonary Trunk and the Aorta. Please consider a sub-analysis to see if using only the Aortic or Left Ventricular ROIs yields a higher correlation with hemoglobin, as these chambers might be more stable at the 30-second time point.

(3) Abstract Clarification: Refine the sentence "mean HU significantly decreased only with NECT" to explicitly state that "mean HU in CECT showed no diagnostic correlation with anemia severity."

(4) Generalizability: Please add a statement in the Discussion emphasizing that the specific ED cutoff values (e.g., 106.25 %EDW) are likely specific to the Philips IQon spectral reconstruction algorithm and may not be directly transferable to dual-source or rapid-kVp switching DECT platforms without cross-calibration.

The manuscript "Evaluation of anemia in non-enhanced and contrast-enhanced dual-energy CT using electron density imaging" presents a scientifically valid and clinically relevant investigation. The use of Electron Density maps to opportunistically screen for anemia in contrast-enhanced studies represents a potentially significant improvement over current HU-based limitations.

However, the current draft suffers from a lack of physical precision regarding the interaction of iodine and electron density, and it fails to meet the data transparency standards of the journal. By releasing the raw data and refining the theoretical explanation to account for the additive nature of iodine density, this work can meet the rigorous standards of PLOS ONE.

Recommendation: Major Revision

**********

6. PLOS authors have the option to publish the peer review history of their article (what does this mean?). If published, this will include your full peer review and any attached files.

If you choose “no”, your identity will remain anonymous but your review may still be made public.

**Do you want your identity to be public for this peer review?**  For information about this choice, including consent withdrawal, please see our Privacy Policy.

Reviewer #1: **Yes:**  Florian Schwarz

**********

[NOTE: If reviewer comments were submitted as an attachment file, they will be attached to this email and accessible via the submission site. Please log into your account, locate the manuscript record, and check for the action link "View Attachments". If this link does not appear, there are no attachment files.]

To ensure your figures meet our technical requirements, please review our figure guidelines: https://journals.plos.org/plosone/s/figures

You may also use PLOS’s free figure tool, NAAS, to help you prepare publication quality figures: https://journals.plos.org/plosone/s/figures#loc-tools-for-figure-preparation.

NAAS will assess whether your figures meet our technical requirements by comparing each figure against our figure specifications.

# Author comment: Author response to Decision Letter 1

**DOI:** 10.1371/journal.pone.0352504.r002

7 Mar 2026

<Authors’ Responses>

Additional Editor Comments:

Thank you for your message and for your patience regarding the review of your manuscript entitled “Evaluation of anemia in non-enhanced and contrast-enhanced dual-energy CT using electron density imaging” (Manuscript ID: PONE-D-25-42920).

We apologize for the delay in reaching a decision. During the peer review process, several invited reviewers declined to evaluate the manuscript, and in a number of instances these declinations were received close to the end of the review invitation period. As a result, additional rounds of reviewer invitations were required, which led to an unavoidable delay in completing the review process.

The peer review has now been completed. After careful evaluation of the reviewers’ reports, I agree with the recommendation that the manuscript requires major revision before it can be considered further for publication in PLOS ONE. In addition to the points raised by the reviewers, I have also identified a small number of minor editorial issues that will need to be addressed in the revision.

E1) Please justify the age 19 and above for selection.

R1) We thank the Academic Editor for these comments. The inclusion criterion of age ≥19 years was used to restrict the study population to adults. This approach ensured consistency with adult reference ranges for hemoglobin and hematocrit and avoided the known physiological variability of hematologic parameters in pediatric populations. We have now added this justification explicitly to the Study Design subsection of the Methods.

[Revision in Materials and Methods]

Study design

This retrospective study was approved by the institutional ethics review board (approval number: 2024AS0179), which granted a waiver of informed consent. No minors participated in the study. We identified 23,023 consecutive patients in our institution’s database who underwent either non-enhanced or contrast-enhanced chest CT using DECT between May 2019 and November 2023. The inclusion criteria were: 1) adults aged ≥19 years, because pediatric patients were excluded to avoid age-related differences in hematologic reference ranges and contrast enhancement characteristics; 2) individuals diagnosed with anemia or healthy individuals with normal Hb levels; and 3) those with Hb, Hct, and RBC count tests performed within 7 days before or after the CT scan.

E2) And all p-value should be written in small p letter.

R2) We carefully revised the entire manuscript to ensure that all p-values are consistently reported using a lowercase “p,” in accordance with journal style requirements.

Comments to the Author

Reviewer #1: This manuscript presents a large-scale, retrospective cohort study investigating the diagnostic utility of Electron Density (ED) maps derived from Dual-Energy Computed Tomography (DECT)—specifically employing dual-layer spectral detector CT (SDCT)—for the opportunistic assessment of anemia. The authors have analyzed a substantial dataset comprising 5,103 adult subjects, stratified into a non-enhanced CT (NECT) cohort of 2,558 patients and a contrast-enhanced CT (CECT) cohort of 2,545 patients. The primary objective was to validate ED as a robust surrogate biomarker for hemoglobin (Hb) and hematocrit (Hct) in the presence of iodinated contrast media, a scenario where conventional CT attenuation (Hounsfield Units, HU) traditionally fails due to the variable enhancement of the blood pool.

The context of this research is rooted in the evolving paradigm of opportunistic screening in radiology. Anemia is a prevalent comorbidity, particularly within hospital-based populations undergoing cross-sectional imaging for oncologic staging, trauma assessment, or acute abdominal pathology. It is well-established that anemia is an independent predictor of adverse outcomes, including increased perioperative mortality, prolonged hospitalization, and reduced quality of life. However, mild to moderate anemia often goes undiagnosed until it becomes symptomatic or severe.

While the manuscript addresses a high-value clinical question and leverages a robust sample size, there are significant reservations regarding the Technical Soundness of the authors' physical interpretation, the rigor of the Statistical Analysis, and compliance with Data Availability requirements. The assertion that ED is "independent" of contrast enhancement is physically imprecise and contradicted by the authors' own data, which shows a measurable shift in ED attributable to iodine. Furthermore, the submission fails to meet the PLOS ONE requirement for providing the "minimal data set" necessary for replication.

Therefore, I recommend a Major Revision.

Major Revisions:

QM1-1) Mandatory Data Availability Update: Your current Data Availability Statement is non-compliant with PLOS ONE policy. Summary tables (S1-S3) are insufficient for replication. You must provide the Minimal Data Set consisting of the individual data points (Age, Sex, Lab Values, CT Values) used to calculate the correlations and ROC curves. Please deposit this de-identified dataset in a public repository (e.g., Dryad, Figshare) or upload it as a supporting information file. The manuscript cannot be accepted without this data.

RM1-1) We fully agree with this important point and thank the reviewer for highlighting it. Accordingly, we provided a Data Set of the study data as supplementary material (S1 File).

• In accordance with the reviewer’s recommendation, we initially intended to provide the complete dataset, including the age variable, to ensure transparency and facilitate reproducibility of the analysis. However, during the editorial technical check, the PLOS ONE Editorial Office identified that the Supporting Information file contained potentially identifying information. Specifically, age was considered personally identifiable information under the journal’s data-sharing policy. Following the guidance from the Editorial Office, we have therefore removed the age variable and any potentially identifying information from the dataset. The revised Supporting Information file now contains only fully anonymized data that comply with the journal’s policies on participant privacy and data sharing. The anonymized dataset has been re-uploaded as the revised Supplementary Material. We hope that this revision addresses the concerns regarding data availability while ensuring compliance with the journal’s ethical and privacy requirements. We greatly appreciate the reviewer’s understanding.

QM1-2) Correction of Physical Interpretation: In the Discussion, you claim that "ED reflects tissue characteristics independently of contrast enhancement." This is contradicted by your own results (Table 2), which show that contrast administration increases the mean ED of non-anemic patients by approximately 1.6 units (from ~105.0 to ~106.6). This shift is larger than the difference between non-anemic and severely anemic patients (~1.2 units).

You must revise the text to acknowledge that iodine DOES contribute to electron density.

Explain that the utility of ED lies in the fact that the variance of this iodine contribution is low enough to preserve the anemia signal, whereas in HU, the iodine variance obliterates the signal.

RM1-2) We thank the reviewer for this critical and well-founded observation and fully agree. We revised the Discussion to correct the imprecise wording and now explicitly acknowledge that iodinated contrast media contribute additively to measured electron density. As the reviewer noted, our data show a measurable upward shift in mean ED after contrast administration. The revised manuscript clarifies that the key advantage of ED over HU in contrast-enhanced CT is not complete independence from iodine, but the lower variance of the iodine-related contribution to ED relative to HU. This lower variance preserves the anemia-related signal, whereas variability in iodine concentration in HU measurements obscures the relationship between attenuation and hemoglobin. This revision aligns the physical interpretation with our empirical findings (Table 2) and strengthens the study’s conceptual framework.

[Revision in Discussion]

Additionally, ED has been reported to be comparable to attenuation in single-energy CT (19, 20). Recent studies have suggested that ED values show no significant correlation with iodine concentration, implying that ED may reflect intrinsic tissue characteristics relatively independently of contrast enhancement (21). Our findings refine this interpretation. Although ED primarily reflects intrinsic tissue composition, our data indicate that iodinated contrast agents contribute additively to measured ED values. This effect is reflected in the systematic upward shift in mean ED in CECT compared with NECT. ED should therefore not be interpreted as completely independent of contrast enhancement. Rather, the diagnostic advantage of ED in CECT appears to arise from the relatively low variance of the iodine-related contribution. Although iodine increases absolute ED values, its variability appears sufficiently constrained to preserve the underlying anemia-related signal. This interpretation is supported by subanalysis across cardiac anatomical sites. Although correlations were lower in the pulmonary trunk and right ventricle than in the aorta and left ventricle, ED values maintained moderate correlations with hematologic parameters across all anatomical sites in CECT. In contrast, HU measurements in CECT are strongly influenced by variability in iodine concentration within the blood pool. This variability overwhelms attenuation differences attributable to Hb concentration and eliminates meaningful correlation with anemia severity. This distinction likely explains why ED, but not HU, retained diagnostic utility in CECT.

QM1-3) Hemodynamic Confounders: Please discuss the limitation of using a fixed 30-second scan delay. In patients with severe anemia, hyperdynamic circulation (high cardiac output) is common. This can lead to faster bolus transit, potentially reducing the iodine concentration in the aorta at 30 seconds compared to non-anemic patients. This hemodynamic variable is a confounder that might artificially lower ED in anemic patients (amplifying your signal) or raise it in heart failure patients (masking the signal).

RM1-3) We are sincerely grateful to the reviewer for this thoughtful and physiologically insightful comment. We agree that the use of a fixed 30-second scan delay introduces a potential hemodynamic confounder, particularly in conditions associated with altered cardiac output. In response, we have revised the Discussion section to explicitly acknowledge this limitation and to clarify the potential directionality of its effects. We further provide several considerations that reduce the likelihood that this confounder solely accounts for our findings: (1) independent validation of the ED–laboratory associations in the non-enhanced CT cohort, where iodine is absent; (2) the large and physiologically heterogeneous contrast-enhanced CT cohort (n = 2,545), which would be expected to introduce non-directional variability rather than systematic bias; and (3) the differential behavior of attenuation values, as HU measurements demonstrated near absence of correlation with laboratory parameters under identical acquisition and contrast conditions. We believe that these revisions improve the methodological transparency of the study and appropriately contextualize the potential influence of hemodynamic variability on our results.

[Revision in Discussion]

In the present study, a fixed 30-second scan delay was used for all contrast-enhanced CT examinations. Cardiac output is a major determinant of contrast bolus kinetics. High-output states—commonly observed in severe anemia as a compensatory mechanism—can accelerate contrast arrival and washout, whereas low-output states, such as heart failure, may delay peak aortic enhancement (25, 26). Consequently, at a uniform 30-second acquisition time, intravascular iodine concentration—and therefore its contribution to measured blood pool ED—may vary systematically according to individual hemodynamic status. In patients with severe anemia and compensatory hyperdynamic circulation, more rapid bolus transit may reduce aortic iodine concentration at the time of imaging (27), thereby directionally reinforcing ED reductions attributable to decreased RBC mass and potentially strengthening the observed associations. In contrast, in patients with reduced cardiac output, delayed peak enhancement may partially offset the expected ED decrease despite the presence of clinically significant anemia. Because cardiac output and related hemodynamic parameters were not available in this retrospective cohort, adjustment for this potential confounder was not feasible.

Nevertheless, several considerations mitigate the potential influence of this hemodynamic confounding. First, the moderate correlations observed between ED and laboratory parameters in the NECT cohort (r = 0.657–0.770). This finding provides independent evidence that ED intrinsically reflects RBC composition and supports the interpretation that the associations observed in the CECT cohort are not merely artifactual. Second, the large size of the CECT cohort (n = 2,545) includes patients with a wide range of physiological and cardiovascular conditions, encompassing both normal and impaired cardiac output states. In a population of this magnitude, inter-individual hemodynamic variability is more likely to contribute to stochastic variability rather than directional bias, thereby tending to dilute, rather than spuriously enhance, the strength of observed correlations. Third, the advantage of ED over conventional HU measurements in the contrast-enhanced setting is highlighted by the near absence of correlation between HU and laboratory parameters. This finding confirms that iodine predominantly determines HU values after contrast administration, rendering HU diagnostically unreliable for anemia detection. In contrast, ED maintained statistically significant and clinically meaningful correlations with Hb, Hct, and RBC count under identical acquisition conditions. The persistence of these associations despite shared exposure to contrast timing and potential hemodynamic variability suggests that ED retains partial functional independence from intravascular iodine concentration, consistent with its underlying physical basis in ED rather than HU. Future prospective studies incorporating bolus-tracking or individualized acquisition timing, along with documentation of hemodynamic variables, would help disentangle iodine-related variability from the intrinsic ED signal associated with RBC composition.

Minor Revisions:

Qm1-1) Bland-Altman Analysis: While Pearson's correlation is useful, it does not measure agreement. To validate the regression equations presented in Table 3 for clinical use, please consider adding a Bland-Altman plot to visualize the bias and limits of agreement between the "Predicted Hb" (derived from ED) and the "Actual Hb."

Rm1-1) We thank the reviewer for this helpful suggestion. We agree that Bland–Altman analysis provides information on agreement beyond correlation alone. Accordingly, we added Bland–Altman plots comparing ED-derived predicted hemoglobin, hematocrit, and red blood cell counts with laboratory-measured values. These results are now provided in a supplementary figure.

During the process of addressing the reviewer’s comments, we also identified and corrected an error in the formulas used in Table 3: the independent variable should have been ED rather than hemoglobin, hematocrit, or red blood cell counts. In addition, to reduce potential confusion, Figure 4 has been revised to display ED on the x-axis rather than the y-axis, and the figure has been redrawn accordingly.

[Revision in Figure 4]

New Figure 4 (A)

New Figure 4 (B)

[Revision in Supplementary Figure 1]

S1 Figure. Bland–Altman plots demonstrating agreement between laboratory-measured hemoglobin, hematocrit, and red blood cell counts and electron density (ED)–derived predicted values. (A) Contrast-enhanced CT. (B) Non-contrast CT.

(A)

(B)

Qm1-2) ROI Specificity: You averaged the ED values from the right and l

Attachment Submitted filename: authors reponses.docx

# Peer review: Decision Letter 1

**DOI:** 10.1371/journal.pone.0352504.r003

**Authors:** M Tanveer Hossain Parash, M Tanveer Hossain Parash

13 Apr 2026

PONE-D-25-42920R1

Evaluation of anemia in non-enhanced and contrast-enhanced dual-energy CT using electron density imaging

PLOS One

Dear Dr. Kim,

Thank you for submitting your manuscript to PLOS ONE. After careful consideration, we feel that it has merit but does not fully meet PLOS ONE’s publication criteria as it currently stands. Therefore, we invite you to submit a revised version of the manuscript that addresses the points raised during the review process.

Please submit your revised manuscript by May 28 2026 11:59PM. If you will need more time than this to complete your revisions, please reply to this message or contact the journal office at plosone@plos.org. When you're ready to submit your revision, log on to https://www.editorialmanager.com/pone/ and select the 'Submissions Needing Revision' folder to locate your manuscript file.

Please include the following items when submitting your revised manuscript:

- A letter that responds to each point raised by the academic editor and reviewer(s). You should upload this letter as a separate file labeled 'Response to Reviewers'.
- A marked-up copy of your manuscript that highlights changes made to the original version. You should upload this as a separate file labeled 'Revised Manuscript with Track Changes'.
- An unmarked version of your revised paper without tracked changes. You should upload this as a separate file labeled 'Manuscript'.

If you would like to make changes to your financial disclosure, please include your updated statement in your cover letter. Guidelines for resubmitting your figure files are available below the reviewer comments at the end of this letter.

If applicable, we recommend that you deposit your laboratory protocols in protocols.io to enhance the reproducibility of your results. Protocols.io assigns your protocol its own identifier (DOI) so that it can be cited independently in the future. For instructions see: https://journals.plos.org/plosone/s/submission-guidelines#loc-laboratory-protocols. Additionally, PLOS ONE offers an option for publishing peer-reviewed Lab Protocol articles, which describe protocols hosted on protocols.io. Read more information on sharing protocols at https://plos.org/protocols?utm_medium=editorial-email&utm_source=authorletters&utm_campaign=protocols.

As the corresponding author, your ORCID iD is verified in the submission system and will appear in the published article. PLOS supports the use of ORCID, and we encourage all coauthors to register for an ORCID iD and use it as well. Please encourage your coauthors to verify their ORCID iD within the submission system before final acceptance, as unverified ORCID iDs will not appear in the published article. *Only*  the individual author can complete the verification step; PLOS staff *cannot*  verify ORCID iDs on behalf of authors.

We look forward to receiving your revised manuscript.

Kind regards,

M Tanveer Hossain Parash, MBBS, M Phil

Academic Editor

PLOS One

Journal Requirements:

If the reviewer comments include a recommendation to cite specific previously published works, please review and evaluate these publications to determine whether they are relevant and should be cited. There is no requirement to cite these works unless the editor has indicated otherwise.

Please review your reference list to ensure that it is complete and correct. If you have cited papers that have been retracted, please include the rationale for doing so in the manuscript text, or remove these references and replace them with relevant current references. Any changes to the reference list should be mentioned in the rebuttal letter that accompanies your revised manuscript. If you need to cite a retracted article, indicate the article’s retracted status in the References list and also include a citation and full reference for the retraction notice.

[Note: HTML markup is below. Please do not edit.]

Reviewers' comments:

Reviewer's Responses to Questions

**Comments to the Author**

1. If the authors have adequately addressed your comments raised in a previous round of review and you feel that this manuscript is now acceptable for publication, you may indicate that here to bypass the “Comments to the Author” section, enter your conflict of interest statement in the “Confidential to Editor” section, and submit your "Accept" recommendation.

Reviewer #1: (No Response)

Reviewer #2: (No Response)

**********

2. Is the manuscript technically sound, and do the data support the conclusions?

The manuscript must describe a technically sound piece of scientific research with data that supports the conclusions. Experiments must have been conducted rigorously, with appropriate controls, replication, and sample sizes. The conclusions must be drawn appropriately based on the data presented.

Reviewer #1: Partly

Reviewer #2: (No Response)

**********

3. Has the statistical analysis been performed appropriately and rigorously?

Reviewer #1: Yes

Reviewer #2: (No Response)

**********

4. Have the authors made all data underlying the findings in their manuscript fully available?

The PLOS Data policy requires authors to make all data underlying the findings described in their manuscript fully available without restriction, with rare exception (please refer to the Data Availability Statement in the manuscript PDF file). The data should be provided as part of the manuscript or its supporting information, or deposited to a public repository. For example, in addition to summary statistics, the data points behind means, medians and variance measures should be available. If there are restrictions on publicly sharing data—e.g. participant privacy or use of data from a third party—those must be specified.

Reviewer #1: No

Reviewer #2: (No Response)

**********

5. Is the manuscript presented in an intelligible fashion and written in standard English?

PLOS ONE does not copyedit accepted manuscripts, so the language in submitted articles must be clear, correct, and unambiguous. Any typographical or grammatical errors should be corrected at revision, so please note any specific errors here.

Reviewer #1: Yes

Reviewer #2: (No Response)

**********

6. Review Comments to the Author

Please use the space provided to explain your answers to the questions above. You may also include additional comments for the author, including concerns about dual publication, research ethics, or publication ethics. (Please upload your review as an attachment if it exceeds 20,000 characters)

Reviewer #1: The authors have addressed Reviewer 1's major concerns substantively. The revised discussion now correctly frames ED as not independent of iodine but rather having low iodine-related variance — a much stronger and more accurate argument. The hemodynamic confounder discussion is thorough. The Bland–Altman plots and site-specific subanalysis were appropriate additions. The formula errors in Table 3 were corrected. Data have been provided as S1 File, though with caveats (see below).

Major Comments

(1) Incomplete minimal dataset (S1 File).

Upon inspection of the supplementary data, the file contains Sex, Hb, and per-site HU/ED values — but I could not identify columns for Hct or RBC count, which are key dependent variables in the correlation and ROC analyses. If these are truly absent, the dataset remains insufficient for full replication of the reported analyses. The authors should confirm and, if necessary, add these variables. Additionally, the removal of age — while understandable on privacy grounds — should be noted as a limitation for reproducibility, since age-stratified analyses cannot be replicated.

(2) Correlation method discrepancy:

The abstract and Table 3 header state "Pearson's correlation coefficient," yet the revised Methods section now states that Spearman's rank correlation was used when normality was not met. It is unclear which method was actually applied for each analysis. The authors should explicitly state in the Results which coefficient was used for each comparison, or provide normality test results.

(3) Clinical utility remains unclear for CECT.

The AUCs for detecting any-grade anemia on CECT are modest (0.73 males, 0.73 females) with sensitivities of 56–70% and specificities of 66–79%. These values are borderline for a screening tool. The Discussion should more explicitly acknowledge that while ED on CECT is statistically significant, its discriminatory performance for mild anemia may not be clinically actionable in isolation. As written, the conclusions somewhat overstate the CECT utility.

Minor Comments

- The manuscript still contains a duplicated sentence in the Limitations paragraph: "First, our DECT scans were conducted using a single specific CT scanner…" followed immediately by "First, our DECT scans were performed on a single-vendor platform…" — the old text was not fully removed (visible in the tracked-changes version, page 66, lines 402–414).

- Hemoglobin units are reported as "mg/dl" throughout, but the standard unit for hemoglobin is g/dL. This should be corrected.

- Figure 3 legend states "(A) contrast-enhanced CT and (B) non-enhanced CT," but the uploaded images appear to show the reverse order based on the ED scale ranges (NECT ~103–106 in Fig 3B; CECT ~105–108 in Fig 3A). The authors should verify figure-label concordance.

- The S4 Table (site-specific CECT correlations) is referenced in the text but was not visible in the supporting materials I received for review. Please ensure it is included.

Reviewer #2: 1. Study Novelty and Clinical Impact

The concept of using CT-derived parameters for anemia detection is not entirely novel, as prior studies have evaluated HU and DECT-derived metrics.

The manuscript needs to better highlight what is new:

Is ED superior to VNC?

Is it clinically implementable in routine workflow?

Currently, the clinical impact is not strongly justified.

Suggestion: Clearly state how ED changes clinical decision-making beyond existing methods.

2. Study Design Limitations (Retrospective Nature)

The study is retrospective with no control over acquisition variability.

The 7-day window between CT and lab values may introduce temporal bias.

Concern: Hemoglobin levels can change rapidly, especially in hospitalized patients.

Suggestion:

Justify the 7-day window

Consider sensitivity analysis (e.g., ≤24–48 hours subgroup)

3. Confounding Variables Not Addressed

Important confounders are missing:

Hydration status

Cardiac output

Renal function

Contrast timing variability

BMI / body habitus

Although hemodynamic confounding is partially discussed, it is not quantified.

Impact: These factors may significantly influence ED and HU values.

4. Statistical Analysis Concerns

Reliance on correlation (r values) is insufficient.

Correlation ≠ clinical agreement.

Issues:

No clear multivariate analysis

No adjustment for confounders

Regression models are not validated externally

Suggestion:

Add multivariate regression

Provide model performance (R², RMSE)

Include internal validation (bootstrap or split-sample)

5. Lack of External Validation

Single-center study using a single scanner (Philips IQon)

ED values are vendor-dependent

Impact: Limits generalizability

Suggestion:

Explicitly emphasize this limitation

Avoid overgeneralizing cutoff values

6. Interpretation of ED and Contrast Effects

The manuscript initially suggests ED is independent of contrast, but data show otherwise.

Even after revision, interpretation remains overstated.

Key issue:

ED is less affected, not independent.

Suggestion:

Use cautious wording such as:

“ED demonstrates relative robustness to contrast variation rather than independen

7. Clinical Applicability is Unclear

How would ED-based anemia detection be used?

Screening?

Opportunistic detection?

Replacement for lab tests?

Concern: No workflow integration discussed

**********

7. PLOS authors have the option to publish the peer review history of their article (what does this mean?). If published, this will include your full peer review and any attached files.

If you choose “no”, your identity will remain anonymous but your review may still be made public.

**Do you want your identity to be public for this peer review?**  For information about this choice, including consent withdrawal, please see our Privacy Policy.

Reviewer #1: **Yes:**  PD Dr. Florian Schwarz

Reviewer #2: No

**********

[NOTE: If reviewer comments were submitted as an attachment file, they will be attached to this email and accessible via the submission site. Please log into your account, locate the manuscript record, and check for the action link "View Attachments". If this link does not appear, there are no attachment files.]

To ensure your figures meet our technical requirements, please review our figure guidelines: https://journals.plos.org/plosone/s/figures

You may also use PLOS’s free figure tool, NAAS, to help you prepare publication quality figures: https://journals.plos.org/plosone/s/figures#loc-tools-for-figure-preparation.

NAAS will assess whether your figures meet our technical requirements by comparing each figure against our figure specifications.

# Author comment: Author response to Decision Letter 2

**DOI:** 10.1371/journal.pone.0352504.r004

25 May 2026

Authors’ response

We thank both reviewers for their thorough and constructive critique of our revised manuscript. We have addressed all comments in detail below. All corresponding revisions have been clearly indicated in the manuscript using the relevant notation numbers (e.g., R1-1, R2-3, etc.) to facilitate the reviewer’s reference.

Reviewer #1: The authors have addressed Reviewer 1's major concerns substantively. The revised discussion now correctly frames ED as not independent of iodine but rather having low iodine-related variance — a much stronger and more accurate argument. The hemodynamic confounder discussion is thorough. The Bland–Altman plots and site-specific subanalysis were appropriate additions. The formula errors in Table 3 were corrected. Data have been provided as S1 File, though with caveats (see below).

Major Comments

Q1-1) Incomplete minimal dataset (S1 File).

Upon inspection of the supplementary data, the file contains Sex, Hb, and per-site HU/ED values — but I could not identify columns for Hct or RBC count, which are key dependent variables in the correlation and ROC analyses. If these are truly absent, the dataset remains insufficient for full replication of the reported analyses. The authors should confirm and, if necessary, add these variables. Additionally, the removal of age — while understandable on privacy grounds — should be noted as a limitation for reproducibility, since age-stratified analyses cannot be replicated.

R1-1) We thank the reviewer for this careful inspection. We confirm that the uploaded S1 File inadvertently omitted the Hct and RBC count columns. We have now added these variables to the dataset. The file now includes: Sex, Hb, Hct, RBC count, mean ED per site, and mean HU per site for both NECT and CECT cohorts. We have also added a sentence to the S1 File description acknowledging that the removal of continuous age data limits full replication of any age-stratified analyses: “Individual-level age data were removed from the supplementary dataset on privacy grounds and replaced with age-group categories; readers wishing to replicate age-stratified subanalyses should be aware of this constraint.”

Q1-2) Correlation method discrepancy:

The abstract and Table 3 header state "Pearson's correlation coefficient," yet the revised Methods section now states that Spearman's rank correlation was used when normality was not met. It is unclear which method was actually applied for each analysis. The authors should explicitly state in the Results which coefficient was used for each comparison, or provide normality test results.

R1-2) We agree with the reviewer’s comment and appreciate the opportunity to clarify this point. In the revised manuscript, we have corrected the description to ensure full consistency between the Abstract and Table 3. Spearman’s rank correlation was the method applied for all primary correlations in Table 3. The wording in the Abstract and the whole main text has been revised to accurately reflect the correlation method applied in each analysis. All corresponding revisions have been indicated in the manuscript using the notation ‘R1-2.’

Q1-3) Clinical utility remains unclear for CECT.

The AUCs for detecting any-grade anemia on CECT are modest (0.73 males, 0.73 females) with sensitivities of 56–70% and specificities of 66–79%. These values are borderline for a screening tool. The Discussion should more explicitly acknowledge that while ED on CECT is statistically significant, its discriminatory performance for mild anemia may not be clinically actionable in isolation. As written, the conclusions somewhat overstate the CECT utility.

R1-3) We thank the reviewer for this insightful comment and fully agree that the discriminatory performance of ED on CECT for detecting any-grade anemia is modest and may not be clinically actionable in isolation, particularly for mild anemia. In response, we have revised the Discussion (including conclusion) to more explicitly address this limitation. Specifically, we now state that the AUCs for detecting any-grade anemia on CECT (0.727 in males; 0.738 in females) represent modest discriminatory performance, especially given that mild anemia constitutes the majority of detected cases, and that sensitivities of 56–70% and specificities of 66–79% at these operating points are unlikely to justify use as a stand-alone screening tool. We further clarify that the primary utility of ED in CECT is best conceptualized as opportunistic detection in patients already undergoing CECT for other indications, with particular relevance for severe anemia, for which AUCs exceeded 0.850–0.895 and sensitivities exceeded 77%. Finally, we emphasize that detection of mild anemia using ED on CECT should be interpreted as supplementary to, rather than a replacement for, laboratory testing.

[Revision in Discussion]

(…)

However, it is important to acknowledge that the AUCs for detecting any-grade anemia on CECT (0.727 in males; 0.738 in females) represent modest discriminatory performance, particularly given that mild anemia constitutes the majority of detected cases. At these operating points, sensitivities of 56–70% and specificities of 66–79% are unlikely to be clinically actionable as stand-alone screening evidence. The primary utility of ED in CECT is therefore best conceptualized as opportunistic detection — the recognition of unexpected or pre-existing anemia in patients already undergoing DECT for an unrelated clinical indication, such as oncology staging, pulmonary embolism workup, or trauma evaluation. This is particularly relevant for severe anemia, for which AUCs exceeded 0.850–0.895 and sensitivities exceeded 77%, representing a clinically meaningful level of discriminatory performance. In this opportunistic context, ED measurement requires no additional scan time, radiation exposure, or contrast administration, as the spectral data are inherently available from the standard acquisition. Automated flagging of reduced blood pool ED values within structured CT report templates or artificial intelligence-based post-processing workflows could prompt clinicians to order confirmatory laboratory testing at an earlier stage than they otherwise might, particularly in oncology patients at elevated risk for treatment-related anemia. Mild anemia detection using ED on CECT should be interpreted as supplementary to, not a replacement for, laboratory testing, and laboratory confirmation remains essential before any clinical decision-making. The feasibility and clinical impact of integrating ED-based anemia flagging into routine imaging workflows warrant prospective evaluation in future studies.

(…)

In conclusion, ED derived from DECT showed strong diagnostic utility for anemia detection on NECT and retained meaningful correlations with hematologic parameters on CECT, though its discriminatory performance for any-grade anemia on CECT was modest and should be interpreted as opportunistic detection rather than a stand-alone diagnostic tool. HU on NECT similarly reflected anemia severity, whereas HU on CECT showed no meaningful correlation with hematologic parameters.

Minor Comments

Q1-4) The manuscript still contains a duplicated sentence in the Limitations paragraph: "First, our DECT scans were conducted using a single specific CT scanner…" followed immediately by "First, our DECT scans were performed on a single-vendor platform…" — the old text was not fully removed (visible in the tracked-changes version, page 66, lines 402–414).

R1-4) We have reviewed the Limitations section and confirm that the duplicate sentence has been removed in the current revised manuscript.

Q1-5) Hemoglobin units are reported as "mg/dl" throughout, but the standard unit for hemoglobin is g/dL. This should be corrected.

R1-5) The reviewer is correct. This is an error that was carried throughout the manuscript. We have corrected all instances of “mg/dl” to “g/dL” throughout the manuscript, including Tables 1 and the anemia definition in the Methods. We apologize for this error. All corresponding revisions have been indicated in the manuscript using the notation ‘R1-5.’

Q1-6) Figure 3 legend states "(A) contrast-enhanced CT and (B) non-enhanced CT," but the uploaded images appear to show the reverse order based on the ED scale ranges (NECT ~103–106 in Fig 3B; CECT ~105–108 in Fig 3A). The authors should verify figure-label concordance.

R1-6) We thank the reviewer for this careful observation. We have re-examined the figure files and confirmed that the ED scale ranges are consistent with the correct assignment: Fig 3A shows CECT data (Mean ED ~104–108; Mean HU ~100–500) and Fig 3B shows NECT data (Mean ED ~102–106; Mean HU ~20–60). The figure panels are therefore correctly labeled as ‘(A) contrast-enhanced CT and (B) non-enhanced CT’ in the legend. No change to the figures or legend is required.

Q1-7) The S4 Table (site-specific CECT correlations) is referenced in the text but was not visible in the supporting materials I received for review. Please ensure it is included.

R1-7) We apologize for the oversight. Upon re-examination, we confirmed that S4 Table was included in the supplementary material file of the original submission; however, its legend had been inadvertently omitted from the Supporting Information section of the main manuscript. We have now added the corresponding legend for S4 Table to the Supporting Information section of the revised manuscript. Additionally, as noted in our response to Q1-2, Spearman’s rank correlation was applied throughout the analysis; accordingly, the correlation coefficient notation in S4 Table has been corrected from r to rs.

S4 Table. Spearman’s rank correlations between hematologic parameters and cardiac CT values by anatomical location in CECT.

Ascending aorta Pulmonary trunk Descending aorta Right ventricle Left ventricle

ED HU ED HU ED HU ED HU ED HU

Hb

rs 0.459 −0.029 0.366 −0.077 0.457 −0.005 0.362 −0.059 0.487 <0.001

Significant level <0.001 0.138 <0.001 <0.001 <0.001 0.792 <0.001 0.003 <0.001 0.982

Hct

rs 0.439 −0.024 0.351 −0.074 0.439 0.001 0.339 −0.063 0.466 0.006

Significant level <0.001 0.222 <0.001 <0.001 <0.001 0.952 <0.001 0.002 <0.001 0.764

RBC count

rs 0.400 −0.058 0.307 −0.103 0.403 −0.027 0.297 −0.090 0.420 −0.026

Significant level <0.001 0.003 <0.001 <0.001 <0.001 0.177 <0.001 <0.001 <0.001 0.183

Note— CECT, contrast-enhanced CT; ED, electron density image; HU, CT attenuation; Hb, hemoglobin; Hct, hematocrit; RBC, red blood cell

Reviewer #2:

Q2-1) Study Novelty and Clinical Impact

The concept of using CT-derived parameters for anemia detection is not entirely novel, as prior studies have evaluated HU and DECT-derived metrics.

The manuscript needs to better highlight what is new:

Is ED superior to VNC?

Is it clinically implementable in routine workflow?

Currently, the clinical impact is not strongly justified.

Suggestion: Clearly state how ED changes clinical decision-making beyond existing methods.

R2-1) We sincerely thank the reviewer for this thoughtful and constructive comment, which has helped us substantially strengthen the manuscript. We fully agree that the clinical impact of ED-based anemia detection requires clearer justification. We would like to respectfully clarify that the present study was not designed to directly compare ED with VNC reconstruction, and we therefore regret that we are unable to make direct claims regarding the superiority of ED over VNC at this time. We agree that a dedicated head-to-head comparison between ED and VNC for anemia detection in the CECT setting would be a highly valuable direction for future research, and we have noted this in the revised manuscript. We would, however, like to highlight an important conceptual distinction: VNC reconstruction mathematically removes the iodine signal entirely, whereas ED retains intrinsic tissue composition information and demonstrates relative robustness to iodine variability—preserving the anemia-related signal arising from RBC composition even under contrast-enhanced conditions. To our knowledge, no prior in vivo study has evaluated ED specifically for anemia detection in the CECT setting, and we believe this represents a meaningful contribution to the field.

Regarding the reviewer’s excellent suggestion to more clearly state how ED changes clinical decision-making beyond existing methods, we would like to draw the reviewer’s attention to the following passage already present in the Discussion: ‘Several studies have shown that VNC images from DECT or photon-counting CT can be used to detect and quantify anemia using CECT scans (12-14, 22). These findings, including ours, are clinically significant for oncology patients, who are particularly susceptible to anemia associated with treatments like chemotherapy or conditions such as cancer-associated bleeding. Given that cancer patients frequently undergo CECT scans for diagnostic or evaluative purposes, the ability to predict serum Hb, Hct, and RBC counts from routine CECT scans and perform early anemia evaluation could greatly aid in the timely detection and management of these complications. Consequently, CT imaging could become an even more valuable diagnostic tool in oncology practice.’

Furthermore, to more explicitly reflect both the clinical utility and limitations of ED-based anemia detection, we have revised the Conclusions as follows: ‘ED derived from DECT showed strong diagnostic utility for anemia detection on NECT and retained meaningful correlations with hematologic parameters on CECT, though its discriminatory performance for any-grade anemia on CECT was modest and should be interpreted as opportunistic detection rather than a stand-alone diagnostic tool. HU on NECT similarly reflected anemia severity, whereas HU on CECT showed no meaningful correlation with hematologic parameters.

We hope these clarifications and revisions adequately address the reviewer’s concerns and that the clinical relevance of our findings is now more clearly communicated. We remain grateful for the reviewer’s valuable guidance in improving the manuscript.

Q2-2) Study Design Limitations (Retrospective Nature)

The study is retrospective with no control over acquisition variability.

The 7-day window between CT and lab values may introduce temporal bias.

Concern: Hemoglobin levels can change rapidly, especially in hospitalized patients.

Suggestion:

Justify the 7-day window

Consider sensitivity analysis (e.g., ≤24-48 hours subgroup)

R2-2) We sincerely thank the reviewer for this constructive suggestion. We fully acknowledge that the retrospective nature of this study and the 7-day window between CT acquisition and laboratory testing represent potential limitations, particularly in hospitalized patients whose hemoglobin levels may fluctuate rapidly. Regarding the justification for the 7-day window: this interval was adopted to maximize cohort size while including patients whose clinical condition was sufficiently stable to allow meaningful correlation between hematologic parameters and CT-derived measurements. A narrower window would have substantially reduced the available sample size. In response to the reviewer’s suggestion, we performed a sensitivity analysis stratifying the cohort by the interval between CT and laboratory testing into three subgroups: less than 24 hours (0-24 hours), less than 48 hours (0-48 hours), and greater than 48 hours only. The results are summarized in the revised manuscript as Supplementary Table S5.

The Spearman’s rank correlation coefficients between mean ED and hematologic parameters were highly consistent across all temporal subgroups. For CECT, the correlations between mean ED and Hb were rs = 0.512 (0-24 hours) and rs = 0.508 (0-48 hours), compared with rs = 0.556 in the full cohort. Similar patterns were observed for Hct and RBC count. For NECT, correlations between mean ED and Hb were rs = 0.724 (0-24 hours) and rs = 0.724 (0-48 hours), compared with rs = 0.756 in the full cohort. Notably, the correlations in the shorter-interval subgroups were essentially equivalent to or slightly weaker than those in the full cohort, confirming that the 7-day inclusion window did not introduce meaningful temporal bias. By contra

Attachment Submitted filename: authors response_final.docx

# Peer review: Decision Letter 2

**DOI:** 10.1371/journal.pone.0352504.r005

**Authors:** M Tanveer Hossain Parash, M Tanveer Hossain Parash, M Tanveer Hossain Parash

11 Jun 2026

Evaluation of anemia in non-enhanced and contrast-enhanced dual-energy CT using electron density imaging

PONE-D-25-42920R2

Dear Dr. Kim,

We’re pleased to inform you that your manuscript has been judged scientifically suitable for publication and will be formally accepted for publication once it meets all outstanding technical requirements.

Within one week, you’ll receive an e-mail detailing the required amendments. When these have been addressed, you’ll receive a formal acceptance letter and your manuscript will be scheduled for publication.

An invoice will be generated when your article is formally accepted. Please note, if your institution has a publishing partnership with PLOS and your article meets the relevant criteria, all or part of your publication costs will be covered. Please make sure your user information is up-to-date by logging into Editorial Manager at Editorial Manager® and clicking the ‘Update My Information' link at the top of the page. For questions related to billing, please contact billing support.

If your institution or institutions have a press office, please notify them about your upcoming paper to help maximize its impact. If they’ll be preparing press materials, please inform our press team as soon as possible -- no later than 48 hours after receiving the formal acceptance. Your manuscript will remain under strict press embargo until 2 pm Eastern Time on the date of publication. For more information, please contact onepress@plos.org.

Kind regards,

M Tanveer Hossain Parash, MBBS, M Phil

Academic Editor

PLOS One

Additional Editor Comments (optional):

Reviewers' comments:

Reviewer's Responses to Questions

**Comments to the Author**

1. If the authors have adequately addressed your comments raised in a previous round of review and you feel that this manuscript is now acceptable for publication, you may indicate that here to bypass the “Comments to the Author” section, enter your conflict of interest statement in the “Confidential to Editor” section, and submit your "Accept" recommendation.

Reviewer #1: All comments have been addressed

Reviewer #2: All comments have been addressed

**********

2. Is the manuscript technically sound, and do the data support the conclusions?

The manuscript must describe a technically sound piece of scientific research with data that supports the conclusions. Experiments must have been conducted rigorously, with appropriate controls, replication, and sample sizes. The conclusions must be drawn appropriately based on the data presented.

Reviewer #1: Yes

Reviewer #2: Yes

**********

3. Has the statistical analysis been performed appropriately and rigorously?

Reviewer #1: Yes

Reviewer #2: Yes

**********

4. Have the authors made all data underlying the findings in their manuscript fully available?

The PLOS Data policy requires authors to make all data underlying the findings described in their manuscript fully available without restriction, with rare exception (please refer to the Data Availability Statement in the manuscript PDF file). The data should be provided as part of the manuscript or its supporting information, or deposited to a public repository. For example, in addition to summary statistics, the data points behind means, medians and variance measures should be available. If there are restrictions on publicly sharing data—e.g. participant privacy or use of data from a third party—those must be specified.

Reviewer #1: Yes

Reviewer #2: Yes

**********

5. Is the manuscript presented in an intelligible fashion and written in standard English?

PLOS ONE does not copyedit accepted manuscripts, so the language in submitted articles must be clear, correct, and unambiguous. Any typographical or grammatical errors should be corrected at revision, so please note any specific errors here.

Reviewer #1: Yes

Reviewer #2: Yes

**********

6. Review Comments to the Author

Please use the space provided to explain your answers to the questions above. You may also include additional comments for the author, including concerns about dual publication, research ethics, or publication ethics. (Please upload your review as an attachment if it exceeds 20,000 characters)

Reviewer #1: This is a large, carefully revised retrospective study testing whether blood-pool electron density (ED) from dual-layer spectral-detector CT reflects anemia severity, on both non-enhanced (NECT, n=2,558) and contrast-enhanced (CECT, n=2,545) chest CT, with laboratory Hb, Hct, and RBC count as the reference standard. The central finding is clinically sensible and well supported: on CECT, conventional attenuation (HU) loses any association with hematologic parameters because iodine dominates the signal, whereas ED retains moderate correlations (rs 0.45–0.58) and useful discrimination for severe anemia (AUC up to ~0.90). On NECT, both ED and HU track anemia well (rs up to 0.77/0.80; AUC up to 0.96). This revision adds age/sex-adjusted partial correlations, bootstrap internal validation, and a CT-to-laboratory interval sensitivity analysis, and now frames the CECT application as opportunistic detection supplementary to laboratory testing. The reference standard is appropriate for anemia, the statistics are rigorous, and the conclusions are now proportionate to the data. I consider the manuscript technically sound and suitable for publication, and I recommend acceptance. The points below are offered only to strengthen the paper and need not hold up acceptance.

1) Shared data and reproducibility

The S1 dataset provides per-patient sex, Hb, Hct, RBC, and site-level HU/ED, so the primary correlations and ROC results are reproducible — this is appreciated. Age was removed, which means the age/sex-adjusted partial correlations and bootstrap confidence intervals (S6, S7) cannot be regenerated from the shared file. If feasible, adding age in a privacy-preserving form (for example, binned by decade) would make those supplementary analyses fully reproducible as well.

2) ROC operating points

The thresholds and their sensitivity/specificity in Table 4 are derived and evaluated in-sample, and the bootstrap was applied to the correlations rather than to the ROC step. A brief note that these are apparent (optimism-uncorrected) values — particularly for the small severe-anemia subgroups (n approximately 145–178) — would calibrate reader expectations. Reporting bootstrap-validated sensitivity and specificity would be a nice optional addition.

3) NECT and CECT overlap

The two groups are drawn from the same institution and period and overlap at the patient level (identical Hb/Hct/RBC triplets appear in both sheets of the dataset). Reporting how many patients contributed both examinations, and softening any implication that the NECT results independently confirm the CECT results, would be more transparent.

4) Positioning and the ED-versus-HU message

The statement that the in-vivo utility of ED for anemia "has not been clearly established" is a little strong: Yang et al. (Quantitative Imaging in Medicine and Surgery, 2025) reported in-vivo ED-based anemia detection on the same dual-layer Philips platform (aortic-arch ED AUC 0.81, n=240, unenhanced), and contrast-enhanced spectral and photon-counting VNC anemia detection are established. Citing this work and re-calibrating the novelty claim around what is genuinely new here — the largest cohort to date, the direct NECT-versus-CECT comparison, and sex-specific severity cutoffs — would pre-empt an easy criticism. Relatedly, on NECT, HU performs at least as well as ED (for example, severe-anemia AUC 0.971 vs 0.956 in females); making explicit that the real advance is the CECT setting, where HU fails and ED survives, would strengthen rather than weaken the paper.

Minor points: (a) R² is computed as the square of a Spearman coefficient (S6/S7) and described as variance explained; relabelling it rs² with a short caveat would be more precise. (b) In S4 Table, the left-ventricle column appears misaligned (HU cells show "<0.001"/"0.982" where a coefficient should appear). (c) A stray ellipsis ends the sentence "...across different imaging environments..." in the limitations; please delete. (d) Figures 3 and 4 are ordered contrast-enhanced before non-enhanced, opposite to the rest of the manuscript; harmonizing would help the reader. None of these affect my recommendation, which is to accept.

Reviewer #2: The manuscript is well conducted, and the authors have adequately addressed most methodological concerns. However, clarification of the incremental value of ED compared with conventional HU measurements, particularly in the NECT cohort, would further strengthen the clinical relevance of the study. Subject to satisfactory revision, the manuscript is suitable for publication.

**********

7. PLOS authors have the option to publish the peer review history of their article (what does this mean?). If published, this will include your full peer review and any attached files.

If you choose “no”, your identity will remain anonymous but your review may still be made public.

**Do you want your identity to be public for this peer review?**  For information about this choice, including consent withdrawal, please see our Privacy Policy.

Reviewer #1: **Yes:**  Florian Schwarz

Reviewer #2: No

**********

# Editor report: Acceptance letter

**DOI:** 10.1371/journal.pone.0352504.r006

**Authors:** M Tanveer Hossain Parash, M Tanveer Hossain Parash, M Tanveer Hossain Parash

PONE-D-25-42920R2

PLOS One

Dear Dr. Kim,

I'm pleased to inform you that your manuscript has been deemed suitable for publication in PLOS One. Congratulations! Your manuscript is now being handed over to our production team.

At this stage, our production department will prepare your paper for publication. This includes ensuring the following:

* All references, tables, and figures are properly cited

* All relevant supporting information is included in the manuscript submission,

* There are no issues that prevent the paper from being properly typeset

You will receive further instructions from the production team, including instructions on how to review your proof when it is ready. Please keep in mind that we are working through a large volume of accepted articles, so please give us a few days to review your paper and let you know the next and final steps.

Lastly, if your institution or institutions have a press office, please let them know about your upcoming paper now to help maximize its impact. If they'll be preparing press materials, please inform our press team within the next 48 hours. Your manuscript will remain under strict press embargo until 2 pm Eastern Time on the date of publication. For more information, please contact onepress@plos.org.

You will receive an invoice from PLOS for your publication fee after your manuscript has reached the completed accept phase. If you receive an email requesting payment before acceptance or for any other service, this may be a phishing scheme. Learn how to identify phishing emails and protect your accounts at https://explore.plos.org/phishing.

If we can help with anything else, please email us at customercare@plos.org.

Thank you for submitting your work to PLOS ONE and supporting open access.

Kind regards,

PLOS ONE Editorial Office Staff

on behalf of

Dr. M Tanveer Hossain Parash

Academic Editor

PLOS One
